# Elimination testing with adapted scoring reduces guessing and anxiety in multiple-choice assessments, but does not increase grade average in comparison with negative marking

**DOI:** 10.1371/journal.pone.0203931

**Published:** 2018-10-02

**Authors:** Jef Vanderoost, Rianne Janssen, Jan Eggermont, Riet Callens, Tinne De Laet

**Affiliations:** 1 Tutorial Services, Faculty of Engineering Science, KU Leuven, Leuven, Belgium; 2 Leuven Engineering and Science Education Centre, KU Leuven, Leuven, Belgium; 3 Centre of Educational Effectiveness and Evaluation, Faculty of Psychology and Educational Sciences, KU Leuven, Leuven, Belgium; 4 Laboratory of Cellular Transport Systems, Faculty of Medicine, KU Leuven, Leuven, Belgium; University of Wuerzburg, GERMANY

## Abstract

**Background and hypotheses:**

This study is the first to offer an in-depth comparison of elimination testing with the scoring rule of Arnold & Arnold (hereafter referred to as elimination testing with adapted scoring) and negative marking. As such, this study is motivated by the search for an alternative for negative marking that still discourages guessing, but is less disadvantageous for non-relevant student characteristics such a risk-aversion and does not result in grade inflation. The comparison is structured around seven hypotheses: in comparison with negative marking, elimination testing with adapted scoring leads to (1) a similar average score (no grade inflation); (2) students expressing their partial knowledge; (3) a decrease in the number of blank answers; (4) no gender bias in the number of blank answers; (5) a reduction in guessing; (6) a decrease in self-reported test anxiety; and finally (7) students preferring elimination testing with adapted scoring over negative marking.

**Methodology:**

To investigate the above hypotheses, this study implemented elimination testing with adapted scoring and negative marking in real exam settings in two courses in a Faculty of Medicine at a large university. Due to changes in the master of medicine the same two courses were taught to both students of the 1^st^ and 2^nd^ master in the same semester. Given that both student groups could take the same exam with different test instructions and scoring methods, a unique opportunity occurred in which elimination testing with adapted scoring and negative marking could be compared in a high-stakes testing situation. After receiving the grades on the exams, students received a questionnaire to assess their experiences.

**Findings:**

The statistical analysis taking into account student ability and gender showed that elimination testing with adapted scoring is a valuable alternative for negative marking when looking for a scoring method that discourages guessing. In contrast to traditional scoring of elimination testing, elimination testing with adapted scoring does not result in grade inflation in comparison with negative marking. This study showed that elimination testing with adapted scoring reduces blank answers and finds strong indications for the reduction of guessing in comparison with negative marking. Finally, students preferred elimination testing with adapted scoring over negative marking and reported lower stress levels in elimination testing with adapted scoring in comparison with negative marking.

## Introduction

A test consisting of multiple-choice questions is a common assessment method in medicine and life sciences programs as well as in other fields, especially for large student groups. Apart from their fast and objective scoring, multiple-choice questions have been shown to have the potential of showing high reliability as well as high content and construct validity [1]. However, as a multiple-choice question presents the students a set of alternatives, including the correct answer, students can obtain the correct answer by guessing. Guessing is often considered undesired behaviour that should not be rewarded nor stimulated. Furthermore, it has been shown that guessing has a negative effect on the reliability of multiple-choice questions [2–4]. A multitude of scoring methods have been proposed that differ in how they accommodate for guessing [5,6]. Apart from traditional single answer multiple-choice questions where students choose one answer, scoring methods that allow to measure partial knowledge have been introduced. Hakstian & Kansup provide an overview of methods that allow assessing partial knowledge [7,8].

One of the most common scoring methods for multiple-choice questions, here named negative marking (negative marking), introduces a penalty for wrong answers in order to discourage guessing. Such a penalty has been shown to improve test reliability [9,10]. The typical penalty in negative marking is conceived to discourage pure random guessing, but no longer fully discourages guessing when students have partial knowledge. Furthermore, due to the penalty, risk-aversion will influence answering patterns and scores of students [10,11]: risk-averse students will leave questions blank, even when their expected score for the question is above zero when they would guess among the remaining alternatives. Espinosa & Gardeazabal [12] showed that negative marking indeed discriminates against risk-averse students, but that this discrimination rather affected students with little knowledge than students with high or average knowledge levels. They concluded that this discrimination is subservient to the reduced measurement error and they even plead for a higher penalty. Others argued that the influence of risk-taking behavior threatens the measurement of actual mastery of domain knowledge [13,14].

Women have been shown to often act more risk-averse than men [15,16] but the impact hereof on multiple-choice exams is contested. On the one hand it is suggested that female students are more likely to leave an answer blank when they are not certain, hereby introducing an undesired disadvantage [12,17–21]. On the other hand, not all studies find an actual significant difference in score between male and female students. For instance, Bond et al. [22] found no statically significant gender differences for two scoring methods with penalty for guessing for a low-stakes test in life sciences. On the contrary, Pekkarinen [21] showed that women perform worse than men in the entrance examination for Finnish universities and that they skipped more questions than optimal for maximizing the probability of acceptance. As factors other than risk-aversion can induce differences between male and female students (e.g., instructions [17], test preparation [23], test anxiety [24], subject area [6], extrinsic rewards or stakes in general [19,25], question difficulty [6,19], and stereotype threat [19]), gender differences should be interpreted and studied with care.

Elimination testing [26–28] allows for assessing partial knowledge as students can indicate for each of the offered alternatives whether they consider it correct or not. When students want to indicate one alternative as the correct answer, they eliminate all but this alternative. If they eliminate fewer alternatives, students can indicate their doubt. Elimination testing rewards partial knowledge (when students do not eliminate the correct answer) while still punishing misconception and guessing (when students eliminate the correct answer). In elimination testing with traditional scoring [22,27–32] answers are scored based on the response to each alternative separately: a reward is given the correct elimination of each distractor and a penalty for the elimination of the correct answer. The latter penalty for misconception is even more severe compared to negative marking [28,32]. Bond et al. [22] showed that elimination testing with traditional scoring does not introduce a gender bias in test scores in life sciences. Moreover, they found that elimination testing with traditional scoring increases student performance and satisfaction and reduces self-reported anxiety. elimination testing with traditional scoring however leads to “grade inflation” with respect to negative marking: it increases the average test score [22,28,31,32]. This is undesirable as a mere change in scoring method would necessitate the examiner to increase test difficulty or to adapt the threshold for passing. Preliminary results [32,33] have shown that elimination testing with the scoring rule of Arnold & Arnold [34] (elimination testing with adapted scoring) avoids grade inflation while still offering the possibility to measure partial knowledge and while still punishing misconception, hereby discouraging guessing.

This study is the first to offer an in-depth comparison of elimination testing with adapted scoring and negative marking. The comparison is structured around seven hypotheses: in comparison with negative marking, elimination testing with adapted scoring leads to (1) a similar average score (no grade inflation); (2) students expressing their partial knowledge; (3) a decrease in the number of blank answers; (4) no gender bias in the number of blank answers; (5) a reduction in guessing; (6) a decrease in self-reported test anxiety; and finally (7) students preferring elimination testing with adapted scoring over negative marking.

To investigate the above hypotheses, this study implemented elimination testing with adapted scoring and negative marking in real exam settings in two courses in a master of medicine at a large university. Due to changes in the master program the same two courses were taught to both students of the 1^st^ and 2^nd^ master in the same semester. Given that both student groups could take the same exam with different test instructions and scoring methods, a unique opportunity occurred in which elimination testing with adapted scoring and negative marking could be compared on real exams. After receiving the grades on the exams, students received a questionnaire to assess their the experiences.

Prior work most related to this study is that of Bond et al. [22], that compared elimination testing with traditional scoring to negative marking (negative marking) in a voluntary, formative assessment of two modules. The present study differs from [22] in several respects. The first difference with the current study is the use of elimination testing with adapted scoring instead of elimination testing with traditional scoring, which should prevent grade inflation. Secondly, the current study uses high-stakes summative assessments, in fact the only and final exam of both courses, while Bond et al. [22] rely on a voluntary, formative assessment. As formative assessment are low stakes, students may exhibit different behavior, especially regarding guessing, in comparison with summative assessments. Thirdly, the current study has a between-group design compared to a within-group design in [22]. Since the current study is conducted in a real exam situation, it was not possible to have students complete the same exam both with elimination testing and negative marking, as was achieved in [22]. As a result we could not directly compare individual students score for elimination and negative marking, while Bond at al. were able to pair the elimination testing with traditional scoring and negative marking answer sheets of one student for the same test [22]. However, by offering students the two scoring procedures for each single-answer multiple-choice questions, their behaviour and reasoning and therefore their answering patterns and the scores on the tests could already be affected by the experimental design. The experimental design of the current study prevents this: a student is subject to one scoring method during an exam. In order to correct for other influences, gender and ability is taken into account when studying the impact of the scoring method. The impact is not only evaluated with respect to performance and student perception as in [22] but this study also investigates differences in answering patterns, especially with regard to partial knowledge and omitted items. Fourthly, Bond at al. [22] used a survey regarding students’ experiences both immediately after the test and after receiving their score. The current study surveys the students only after they received their official exam score. As long-term satisfaction is considered more important than short-term, this might be considered a minor disadvantage. Finally, the population in our study consists of 683 1^st^ and 2^nd^ year master students with prior experience in multiple-choice exams with negative marking. This is a considerably larger and more experienced group than the 176 level 1 and level 2 bachelor students of [22].

## Materials and methods

### Ethical statement

Formal permission was obtained from the program advisory committee of the Master of Medicine, in which students, teachers, and teaching assistants are represented. A focus group with student representatives was organized to inform students about the test design and to obtain explicit consent. Students were informed about the data collection and the study at the start of the questionnaire in order to provide written consent for coupling their questionnaire data to their grades As this study concerns an evaluation of assessment practices as part of an educational innovation project, no additional ethical approval from the ethical commission was required on top of the consent of the program advisory committee of the master of medicine.

### Context, test design, available data & methodology

The study was conducted at KU Leuven, Flanders, Belgium. The students are 1^st^ and 2^nd^ year students in the master of Medicine. All students had prior experience with multiple-choice exams, which were scored using negative marking.

## Exams test design

Thanks to changes in the master of Medicine, resulting from the reduction in duration from 4 to 3 years, a unique test design was accomplished. In the “old” master program Paediatrics and Gynaecology-Obstetrics (hereafter referred to as Gynaecology) were taught, in Dutch, in the second year, while in the “reformed” master program these two courses were taught in the first year. Therefore, in the academic year of 2015–2016, the first year of the reform, both first year students of the “old” master program and second year students of the “reformed” master program followed both courses in the second semester. For brevity, we refer to these two groups as the 1^st^ master and 2^nd^ master students. Each course had two examination moments of which students had to attend one (or none). In the first stage the majority of the students are assigned to one of the two examination moments for each course, but afterwards all students can still freely change examination moments. Therefore, students are not randomly distributed between both examination moments. Additionally and obviously, each examination moment had a different set of multiple-choice questions. Table 1 presents the test design of the study. Each exam was administered once with elimination testing with adapted scoring and once with negative marking. Both student groups were exposed once to elimination testing with adapted scoring and once to negative marking. For Paediatrics 1^st^ master students were assessed with elimination testing with adapted scoring, while the 2^nd^ master students were assessed using negative marking. The reverse was done for Gynaecology: 1^st^ master students were assessed using negative marking and 2^nd^ master students using elimination testing with adapted scoring. The exams of Paediatrics and Gynaecology consisted of 40 and 80 questions, respectively.

**Table 1:** Exams test design and number of selected students by course, master/scoring method, examination method and gender.

course	master	scoring method	examination moment	number of students	gender	
					male	female
**Paediatrics**	**1**^**st**^ **master**	**elimination testing with adapted scoring**	**T1**	168	79 (42%)	89 (58%)
			**T2**	179	89 (49%)	90 (51%)
	**2**^**nd**^ **master**	**negative marking**	**T1**	217	89 (41%)	128 (59%)
			**T2**	119	57 (48%)	62 (52%)
**Gynaecology**	**1**^**st**^ **master**	**negative marking**	**T1**	168	79 (42%)	89 (58%)
			**T2**	179	89 (49%)	90 (51%)
	**2**^**nd**^ **master**	**elimination testing with adapted scoring**	**T1**	217	89 (41%)	128 (59%)
			**T2**	119	57 (48%)	62 (52%)

Test administration design for comparing negative marking and elimination testing with adapted scoring. The number of 1^st^ and 2^nd^ master students and their gender is indicated for the two examination moments of each course.

In total, 784 unique students participated in at least one of the four examination moments. 94.5% of the students combined the first examination moments of both courses (T1 Paediatrics June 7^th^, T1 Gynaecology June 13^th^) or the second examination moments of both courses (T2 Paediatrics June 16^th^, T2 Gynaecology June 23^th^). Since differences in exam difficulty and student population might arise between both examination moments, the dataset is further reduced to the 683 students who took an exam for both courses with the two most common examination moment combinations. **Table 1** shows the resulting subsets including their distribution by gender.

Students had four hours to complete each exam. In the week before the examination period an information session was organized to explain the two scoring methods (negative marking and elimination testing with adapted scoring) and the test design to students. As elimination testing with adapted scoring was a new scoring method for the students, additional instructions were provided in a specific lecture and on the virtual learning environment, including a link to a web page with further explanations and an online practicing module.

For all exams, students received the exam questions in a paper booklet. They had to submit their final answers on a specific answer sheet. During the exams with elimination testing with adapted scoring, each student received a summary with a short explanation of the scoring method. After the exams, the answer sheets were digitized using the Remark Office OMR software and automatically graded using software that was developed in-house (publically available on https://github.com/tdelaet/verwerkingMeerkeuzeEliminatie_python).

Summarizing, given the exam test design the following data were available for each student: master level (1^st^ or 2^nd^ master), examination moment for Paediatrics and Gynaecology, exam scores for Paediatrics and Gynaecology, and for each question: score, answering pattern, and knowledge level (Section 2.3 defines the answering patterns and knowledge levels for negative marking and elimination testing with adapted scoring). Additionally, as both gender and ability could influence the exam score, the answering patterns, and the knowledge levels, gender, and grade point average of each student was retrieved from the university data base. This study uses grade point average of the entire academic year (without resits) as a measure for student ability [35].

### Questionnaire

After receiving their grades, a link to an online questionnaire was sent to the students’ university e-mail addresses. A reminder was sent after one week and after 2 weeks. Students were asked to rate whether or not they agreed with the statements on a 5-point Likert scale ranging from “strongly disagree” to “strongly agree”. S1 Table provides the complete list of statements. Most statements were reused from the study of Bond et al. [22], who developed a questionnaire to compare students’ perceptions regarding negative marking and elimination testing with traditional scoring. This study relies on this questionnaire to assess the self-reported test anxiety of students. The questionnaire contains several inverse and complementary statements to detect any bias due to the statement wording. The overall response rate was 26.8%. Table 2 shows the response rate for the different examination moments, including the gender distribution. T-tests showed that grade point average and exam scores of student on the elimination testing with adapted scoring and negative marking exams did not significantly differ from the entire student sample (683 students) neither for the overall population (p-value grade point average 0.13, score elimination testing with adapted scoring 0.36, and score negative marking 0.14) nor for the different examination moments Summarizing, from the questionnaire the responses of a representative student sample on all survey questions were available and could be linked to the exam results.

**Table 2:** Response rate on online questionnaire per examination moment.

master	course & scoring method	examination moment	all students	gender	
				male	female
**1**^**st**^ **master**	**Paediatrics = elimination testing with adapted scoring** **Gynaecology = negative marking**	**T1**	60 (35.7%)	38.0%	33.7%
		**T2**	47 (26.3%)	23.6%	28.9%
**2**^**nd**^ **master**	**Paediatrics = negative marking** **Gynaecology = elimination testing with adapted scoring**	**T1**	56 (25.8%)	29.2%	23.4%
		**T2**	20 (16.8%)	15.8%	17.7%

Response rate for the online questionnaire divided over the different examination moments for the 683 students of the sample studies in this paper specified in Table 1.

### Statistical analyses

Descriptive statistics and statistical analysis using *t-*tests, multi-way ANOVA, and regression models were used to study the influence of the scoring method on the exam score and answering patterns and its interplay with other factors like gender, examination moment, and grade point average. Due to the “crossed” test design (for Paediatrics the 1^st^ master students use elimination testing with adapted scoring and the 2^nd^ master students negative marking; while this is the reverse for Gynaecology), a careful statistical analysis is required. The two courses Paediatrics and Gynaecology are analyzed separately such that if the analysis of both courses are consistent, the effects can be attributed to the scoring method and not to the master level. However, when interpreting the course by course analyses, care should be taken as the factor scoring method in fact encompasses both master level and scoring method (Table 1).

For the questionnaire, non-parametric one-sample *t*-tests were performed on the responses to assess whether the average response was significantly different from neutral. To this end, the 5-point Likert scale was converted to a numeric scale as follows: (5) Strongly agree, (4) Agree, (3) Neither agree nor disagree; (2) Disagree, (1) Strongly disagree. To assess whether the answers were significantly different between male and female students a Wilcoxon signed-rank test was used. All analyses were performed using R.

### Scoring methods

Negative marking and elimination testing with adapted scoring are discussed below for an exam with *N* questions and *n* given response alternatives. Each item has one correct answer and *n-1* distractors. As the exams discussed in this paper use five possible answers, the examples are elaborated for *n = 5*.

#### Negative marking

**Fig 1:**
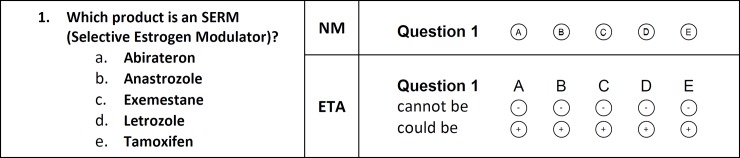
Example of a multiple-choice question for negative marking and elimination testing with adapted scoring with five alternatives (n = 5).

For multiple-choice questions with negative marking, students either indicate the alternative they believe to be correct or leave the question blank. Fig 1 shows an example question for negative marking with five alternatives.

In negative marking, a correct answer receives a score of 1, a wrong answer receives a penalty of $\frac{-1}{n-1}$, a blank answer is scored neutrally (0). Table 3 shows the two answering patterns a student can use in negative marking (indicate one alternative or leave the question blank) for a question with five alternatives, and the scores that can result from the answering patterns. With negative marking three “knowledge levels” can be assessed: full knowledge (student indicates one alternative, which is the correct answer), misconception (student indicates one alternative, which is one of the distractors), or no knowledge.

**Table 3:** Answering patterns, knowledge levels, and corresponding scores for negative marking and elimination testing with traditional or adapted scoring for five alternatives (n = 5) with A the correct answer.

answering pattern	[A B C D E]	negative marking		elimination testing		
		score	knowledge level	scoring		knowledge level
				traditional	adapted	
no doubt	[1 0 0 0 0]	1	full knowledge	1	1	full knowledge
	[0 0 1 0 0]	−1/4	misconception	−1/4	−1/4	partial misconception 1
doubt two	[1 1 0 0 0]	-	-	3/4	3/8	partial knowledge 1
	[0 1 1 0 0]	-	-	−2/4	−1/4	partial misconception 2
doubt three	[1 1 0 1 0]	-	-	2/4	1/6	partial knowledge 2
	[0 1 1 1 0]	-	-	−3/4	−1/4	partial misconception 3
doubt four	[1 1 0 1 1]	-	-	1/4	1/16	partial knowledge 3
	[0 1 1 1 1]	-	-	−1	−1/4	total misconception
blank	[0 0 0 0 0]	0	no knowledge	0	0	no knowledge

The [A B C D E] column provides an example of the answering pattern: 1 corresponds to an alternative indicated by the student (as “could be” in elimination testing), while 0 corresponds to an alternative not indicated by the student (indicated as “cannot be” in elimination testing).

#### Elimination testing

For multiple-choice questions with elimination testing (ET) students indicate the alternatives they believe to be incorrect [27]. Fig 1 shows an example for ET with five alternatives. Students can indicate doubt (partial knowledge) by eliminating fewer than n-1 alternatives. While elimination testing normally only uses one row of “bubbles” (labelled “cannot be”) on which students can indicate the answers they want to eliminate, this study used two rows. On the second row (labelled “could be”), students can indicate the answers they want to keep. Ideally this should be the inverse of the top row. By using both rows, an additional check is provided for the automatic scoring. If the automatic scoring observes that both or none of the two bubbles of an alternative are colored, a manual intervention is triggered.

The most common scoring procedure applied with elimination testing [22,30,31] scores the different alternatives separately: each correctly eliminated alternative leads to a reward of $\frac{1}{n-1}$, while eliminating the correct answer leads to a penalty of -1. Elimination testing with traditional scoring results in a maximum score of +1 and a maximum penalty of -1, with different scores for different levels of partial knowledge and partial misconception (Table 3). It has been shown that elimination testing with traditional scoring results in an increased average score compared with negative marking [22,28,31,32] and in [26] (for general knowledge and mathematical reasoning questions, but not for figural reasoning and general reasoning).

Arnold and Arnold [34] introduced an alternative scoring procedure for elimination testing that rewards partial knowledge but equally penalizes any form of misconception (i.e., eliminating the correct answer) and that allows the examiner to set the penalty associated with this misconception. This study uses a penalty that equals the penalty with negative marking, (i.e., $\frac{-1}{n-1}$), resulting in the scoring method for elimination testing used in this study (elimination testing with adapted scoring). Such a penalty is considered “fair” as it results in an expected gain of zero when guessing[34]. An answer without misconception receives a mark that depends on the number of distractors eliminated (x):

$$score(x)=\frac{x}{\left(n-x)(n-1\right)}.$$

As a result, an answer where all distractors are eliminated (and the correct answer is not) receives a mark of 1; an answer where none of the alternatives are eliminated is scored neutrally (0); partial knowledge receives a partial mark that depends on the number of correctly eliminated distractors (x). Table 3 shows the possible answering patterns a student can give in elimination testing with adapted scoring (ranging from no doubt over doubt between two, three or four alternatives, to full doubt), and the resulting scores depending on which alternative is the correct answer for n = 5. With elimination testing with adapted scoring (2n-1) “knowledge levels” can be measured including full knowledge, different levels of partial knowledge and partial misconception, total misconception, or no knowledge. Interestingly the partial mark a student can receive is always lower than ½. Additionally, students that honestly report their partial knowledge receive a score equal to the expected score when guessing (assuming equal doubt between the remaining alternatives). For instance: students that eliminate three $\left(\frac{n+1}{2}\right)$ of the wrong alternatives receive a score of $\frac{3}{8}\left(\frac{3}{\left(n-3)(n-1\right)}\right)$. If these students would decide to “guess” and indicate one of the remaining alternatives there is a chance of 1/2 to indicate the right (+1) alternative and a chance of 1/2 to indicate the wrong $\left(\frac{-1}{4}(\frac{-1}{n-1})\right)$ alternative, leading to an expected score of $\frac{3}{8}\left(\frac{3}{8}=\frac{1}{2}*1+\frac{1}{2}*\frac{-1}{4}\right)$ or in general $\frac{3}{\left(n-3)(n-1\right)}\left(\frac{3}{\left(n-3)(n-1\right)}=\frac{1}{2}*1+\frac{1}{2}*\frac{-1}{n-1}\right)$. As students receive the expected score according to their knowledge level, the need for guessing diminishes. Students are however still free to determine their own strategy and they can still guess or skip the question.

An interesting observation regarding elimination testing with adapted scoring is that if students leave the question blank (eliminate no alternatives) or do not show partial knowledge (eliminate all but one alternative), the scoring is exactly the same as in negative marking.

## Results

### Exam score and answering patterns

The goal of this section is to study the influence of the scoring method on the exam score (hypothesis 1) and the answering patterns (hypotheses 2–5). In order to select an appropriate statistical methodology, a prior analysis regarding the influence of background variables (master level, gender, and ability) is needed.

#### Univariate statistical analysis

A naive univariate statistical analysis consists of directly comparing the scores and answering patterns between negative marking and elimination testing with adapted scoring for each of the examination moments. Table 4 presents the results of such a naive univariate analysis for the average exam score using a t-test test for hypothesis 1: in comparison with negative marking, elimination testing with adapted scoring leads to a similar average score (no grade inflation).

**Table 4:** Average score and standard deviation for different examination moments for both elimination testing with adapted scoring and negative marking.

course	master	scoring method	examination moment	score	F-test *F*(*p*)	t-test *t*(*p*)
**Paediatrics**	**1**^**st**^ **master**	**elimination testing with adapted scoring**	**T1**	14.98 (2.05)	0.907 (0.510 ^ns^)	-0.673 (0.502 ^ns^)
			**T2**	14.07 (2.25)	0.670 (0.016*)	3.031 (0.003**)
	**2**^**nd**^ **master**	**negative marking**	**T1**	15.13 (2.15)		
			**T2**	13.15 (2.75)		
**Gynaecology**	**1**^**st**^ **master**	**negative marking**	**T1**	13.17 (2.63)	0.909 (0.507 ^ns^)	-4.935 (<0.001***)
			**T2**	12.05 (3.22)	1.558 (0.007**)	-4.635 (<0.001***)
	**2**^**nd**^ **master**	**elimination testing with adapted scoring**	**T1**	14.53 (2.76)		
			**T2**	13.68 (2.58)		

Average grade and standard deviation for the different exams, examination moments, and scoring methods (negative marking and elimination with adapted scoring). Additionally the result of the t-tests for the hypothesis “in comparison with negative marking, elimination testing with adapted scoring leads to a similar average score (no grade inflation)”. The *t*-tests and *F*-tests are done between the different scoring methods (negative marking vs elimination testing with adapted scoring) for the same examination moments. Depending on the result of the *F*-test a two-sided *t*-test for equal or unequal variances was used. Superscripts indicate levels of significance using the following coding

ns *p* > 0.05

* *p* < 0.05

** *p* < 0.01

*** *p* < 0.001.

This analysis does not lead to any clear conclusion concerning the first hypothesis. This analysis assumes that students in the different examination moments and using the two scoring methods have similar characteristics whereas factors like ability, master level, and gender may also influence the score. Therefore, the remainder of this section is devoted to building an appropriate multivariate model for testing the impact of the scoring method while taking into account these factors.

First, due to the crossed test design (Table 1), students exposed to the different scoring methods in both exams have a *different master level*, i.e. they in a different master year. However, as the test design is “crossed” (for Paediatrics the 1^st^ master students use elimination testing with adapted scoring and the 2^nd^ master students have Gynaecology; while this is the reverse for Paediatrics), conclusions hold if they are consistent for both courses (Gynaecology and Paediatrics).

Secondly, *gender* is also a factor that may influence exam scores and answering patterns (hypothesis 4). Table 5 shows that female students score systematically higher for Paediatrics and Gynaecology for both examination moments. Table 1 already showed that the distribution of gender over the masters and examination moments is balanced between examination moments and scoring methods.

**Table 5:** Average score and standard deviation for different test moments and gender using scoring methods negative marking and elimination with adapted scoring.

course	master	scoring method	examination moment	gender	
				male	female
**Paediatrics**	**1**^**st**^ **master**	**elimination testing with adapted scoring**	**T1**	14.81 (2.25)	15.14 (1.85)
			**T2**	13.94 (2.49)	14.20 (2.00)
	**2**^**nd**^ **master**	**negative marking**	**T1**	14.81 (2.07)	15.35 (2.19)
			**T2**	12.66 (2.93)	13.60 (2.52)
**Gynaecology**	**1**^**st**^ **master**	**negative marking**	**T1**	14.26 (3.16)	14.77 (2.34)
			**T2**	13.44 (3.11)	13.92 (1.91)
	**2**^**nd**^ **master**	**elimination testing with adapted scoring**	**T1**	12.80 (2.49)	13.42 (2.49)
			**T2**	11.29 (3.46)	12.75 (2.83)

Average score and standard deviation for the different exams (master level / scoring method, examination moments, gender and scoring methods (negative marking and elimination with adapted scoring)

Thirdly, the *examination moment* may influence the exam scores. For each course two examination moments were organized with different multiple-choice questions. Although both examination moments cover the same topics and use the same number of questions, exam difficulty may differ. Table 4 shows that for both Paediatrics and Gynaecology the average score of students participating in the first examination moment is significantly higher than of students participating in the second examination moment. Moreover, as students could freely select the examination moment in which they participated, a selection bias among the two exam moments may be induced for both exams as (some) students may deliberately chose the first moment and others the second.

Fourthly, and in fact most importantly, the ability of students influences the exam score and the answering patterns. This study uses grade point average as a measure for the student’s ability. S1 Fig illustrates the strong and approximately linear relation between grade point average and exam score (R^2^ = 0.544 for Paediatrics and R^2^ = 0.680 for Gynaecology). Therefore, when analyzing the influence of the scoring method on the score of students and the answering patterns, it is necessary to correct for grade point average. Furthermore, it is important to check if the average grade point average differs between master level, examination moments, and male and female students. Table 6 shows that the average grade point average of students is different for the two examination moments (higher grade point average for students in the first examination moments), the 1^st^ and 2^nd^ master students (higher grade point average for 1^st^ master students), and male and females students (higher grade point average for female students). A multi-way ANOVA (continuous variable grade point average and factors gender, master, and examination moments) confirms that these findings are statistically significant (Table 7). Furthermore, it exposes an interaction effect between examination moment and master level (the first examination moment effect is higher for 2^nd^ master students).

**Table 6:** Grade point average over different examination moments and master.

master	examination moment	gender	
		male	female
**1**^**st**^ **master**	**T1**	72.09 (8.84)	72.11 (8.16)
	**T2**	70.73 (9.12)	72.13 (9.66)
**2**^**nd**^ **master**	**T1**	69.63 (9.48)	71.15 (9.66)
	**T2**	64.82 (12.58)	68.53 (9.40)

The average grade point average (%) and the standard deviation between brackets of male and female students from the 1^st^ and 2^nd^ master participating to the different examination moments.

**Table 7:** Multi-way ANOVA between grade point average and factors examination moment (EM), gender and master level.

response: grade point average	*F*	*p*
**examination moment**	7.95	0.005**
**gender**	4.45	0.035*
**master**	17.07	<0.001***
**examination moment:gender**	1.47	0.226^ns^
**examination moment:master**	4.35	0.037*
**gender:master**	1.66	0.198^ns^
**examination moment:gender:master**	0.08	0.781^ns^

ANOVA Table for Type II tests. ANOVA model: grade point average ~ examination moment * gender * master. Degrees of freedom (numerator, denominator) for all factors: (1, 675). Superscripts indicate levels of significance using the following coding

ns *p* > 0.05

* *p* < 0.05

** *p* < 0.01

*** *p* < 0.001.

The above analysis indicates that when studying the influence of scoring methods in the proposed test design, a regression analysis is needed of the exam score (or answering patterns) and the influencing factors grade point average, gender, and examination moment, interaction factors with grade point average (grade point average*scoring method, grade point average*gender, grade point average*examination moment, grade point average*scoring method*examination moment, see Table 7), and an interaction factor between gender and scoring method (gender*scoring method). The latter effect is added to the model as previous studies have indicated that scoring methods may affect male and female students differently, e.g., due to their risk-aversion [17,18].

#### Hypothesis 1: Grade inflation

The first hypothesis investigates the effect of the scoring method on the exam score. Table 4 shows the average exam scores for the different examination moments. Based on the findings of the previous section, the following multiple linear regression model is constructed for both Paediatrics and Gynaecology to test the first hypothesis:

score∼GPA+SM+gender+EM+GPA*SM+GPA*gender+GPA*EM+GPA*SM*EM+SM*gender.

In this equation, SM is the scoring method and EM the examination moment.

The results of the regression, shown in Table 8, indicate that the factors explained 60% of the variance for Paediatrics (adjusted *R*^2^ = 0.600, residual standard error 1.516, *F*(10,672) = 102.9, *p* < 0.001) and 71% for Gynaecology (adjusted *R*^2^ = 0.706, residual standard error 1.557, *F*(10,672) = 165, *p* < 0.001). The models show that, as expected, grade point average significantly predicts exam score. For Paediatrics, the examination moment also predicts exam score, showing that the scores on the second examination moment are significantly lower. For Gynaecology, gender and the interaction effect between grade point average and gender also predict exam score. This shows that female students score significantly higher on Gynaecology compared to male students and that the impact of gender depends on grade point average: the difference in score between female and male students is larger for students with low grade point average. For both Paediatrics and Gynaecology, no significant effect is found for the scoring method. Therefore, the first hypothesis is accepted: there are no significant differences in average scores between students using negative marking and students using elimination testing with adapted scoring.

**Table 8:** Results of multiple linear regression for predicting exam score.

	Pediatrics			Gynaecology		
	*β*	*t*(672)	*p*	*Β*	*t*(672)	*p*
Intercept	2.635 (1.101)	2.393	0.017*	-5.187 (0.958)	-5.414	<0.001***
grade point average	0.170 (0.015)	11.230	<0.001***	0.258 (0.014)	18.929	<0.001***
SM [negative marking]	1.260 (1.265)	0.996	0.320^ns^	0.440 (1.305)	0.337	0.736^ns^
gender [female]	-0.300 (0.932)	-0.321	0.748^ns^	3.377 (0.927)	3.644	<0.001***
examination moment (EM) [T2]	-2.974 (1.424)	-2.088	0.037*	-0.766 (1.194)	-0.642	0.521^ns^
GPA*SM	-0.013 (0.018)	-0.752	0.452 ^ns^	0.007 (0.018)	0.396	0.692^ns^
GPA*gender	0.006 (0.013)	0.488	0.628^ns^	-0.044 (0.013)	-3.317	0.001***
GPA*EM	0.031 (0.020)	1.548	0.122^ns^	0.009 (0.017)	0.507	0.613^ns^
GPA*SM*EM	0.011 (0.026)	0.410	0.682^ns^	-0.003 (0.026)	-0.129	0.897^ns^
SM*gender	0.33 (0.236)	0.563	0.574^ns^	0.071 (0.243)	0.293	0.769^ns^

The table shows the regression coefficients (β) and the standard deviation between brackets for both the courses (Paediatrics and Gynaecology) (N = 683, R^2^ = 0.600 for Paediatrics and 0.706 for Gynaecology). GPA = grade point average, SM = scoring method, EM = examination moment. Superscripts indicate levels of significance using the following coding

ns *p* > 0.05

* *p* < 0.05

** *p* < 0.01

*** *p* < 0.001.

#### Hypothesis 2: Partial knowledge expression

Table 9 and Table 10 report the answering patterns (defined in Table 3 for negative marking and elimination testing with adapted scoring, respectively) that students used on the two examination moments of Paediatrics and Gynaecology using respectively the percentage of students using the answering patterns on at least one question (Table 9) and the average of the number of questions for which a student used each answering patterns (Table 10).

**Table 9:** Percentage of students that show different answering patterns on at least one multiple-choice question.

			no doubt			blank			doubt		
	scoring method (master)	exam mo-ment	tot	male	female	tot	male	female	tot	male	female
**PED**	**ETA (1**^**st**^**)**	**T1**	100	100	100	50.3	55.6	44.9	97.2	95.6	98.9
		**T2**	100	100	100	31.6	24.1	38.3	95.5	95.2	95.7
	**negative marking (2**^**nd**^**)**	**T1**	100	100	100	71.1	73.2	69.6	-	-	-
		**T2**	100	100	100	81.6	85.7	77.5	-	-	-
**GY**	**negative marking (1**^**st**^**)**	**T1**	100	100	100	93.1	88.5	97.1	-	-	-
		**T2**	100	100	100	91.6	88.8	94.6	-	-	-
	**ETA (2**^**nd**^**)**	**T1**	100	100	100	65.6	61.8	68.6	97.2	96.4	97.9
		**T2**	100	100	100	76.8	75.0	78.5	98.4	96.7	100

ETA is the abbreviation for elimination testing with adapted scoring, PED abbreviates Pediatrics, and GY Gynaecoloy. The answering patterns are defined in Table 3. Doubt is an aggregation of doubt two, doubt three, and doubt four. The full details for the different doubt answering patterns is available in S2 Table and S3 Table.

**Table 10:** Average number of questions for which a student showed each answering pattern.

	scoring method (master)	exam moment	no doubt		blank		doubt	
			male	female	male	female	male	female
**PED**	**ETA (1**^**st**^**)**	**T1**	33.62 (4.68)	33.75 (3.93)	0.42 (0.89)	0.71 (1.23)	5.96 (4.50)	5.54 (3.49)
		**T2**	33.12 (4.42)	32.90 (3.97)	0.78 (0.96)	0.83 (1.19)	6.10 (4.20)	6.27 (3.53)
	**negative marking (2**^**nd**^**)**	**T1**	37.60 (2.41)	37.86 (2.32)	2.40 (2.41)	2.14 (2.32)	-	-
		**T2**	37.19 (2.58)	37.03 (2.78)	3.28 (2.73)	2.84 (2.74)	-	-
**GY**	**negative marking (1**^**st**^**)**	**T1**	73.41 (5.68)	71.60 (5.92)	6.59 (5.68)	8.40 (5.92)	-	-
		**T2**	72.63 (6.12)	71.48 (6.70)	7.37 (6.12)	8.52 (6.70)	-	-
	**ETA (2**^**nd**^**)**	**T1**	63.73 (11.66)	65.05 (10.68)	2.81 (3.67)	2.55 (3.41)	13.46 (9.96)	12.41 (8.97)
		**T2**	60.16 (10.64)	62.65 (9.44)	4.25 (4.90)	3.29 (4.87)	15.60 (8.99)	14.06 (7.04)

Average number of questions for which a student showed each answering pattern and standard deviation (between brackets). ETA is the abbreviation for elimination testing with adapted scoring, PED abbreviates Pediatrics and GY Gynaecoloy. Paediatrics exams have 40 questions and Gynaecology exams have 80 questions. The answering patterns are defined in Table 3. Doubt is an aggregation of doubt two, doubt three, and doubt four. The full details for the different doubt answering patterns is available in the S2 Table and S3 Table.

Table 9 shows that between 95.5% and 98.4% of the students per examination moment express *doubt* between two, three, or four alternatives on at least one question. Students do this on average for between 14.7% and 18.7% of the questions (Table 10). The full details for the different doubt answering patterns is available in S2 Table and S3 Table. Students mainly doubt between two alternatives: between 90.5% and 97.5% of students use this answering pattern at least once and student do this on average for between 5.6% and 12.8% of the questions. These results confirm the second hypothesis: “Students use elimination testing with adapted scoring to express their partial knowledge”.

#### Hypothesis 3–4: Decrease in blank answers and gender bias

Elimination testing with adapted scoring reduces the percentage of students using *blank answers* and the average percentage of blank answers per student in comparison with negative marking. The reduction in percentage of students that leave at least one question blank in elimination testing with adapted scoring with respect to negative marking ranges from 14.8% to 50.0%, with an average reduction of 28.3% (Table 9). The reduction in the average percentage of blank answers per student in elimination testing with adapted scoring with respect to negative marking ranges from 3.9% to 6.4%, with an average reduction of 5.3% (Table 10).

To determine whether this reduction is statistically significant when taking into account gender and grade point average, a multiway ANOVA analysis was performed (Table 11). Since the dataset contains a lot of zero values for the number of blank answers per student, a linear regression model cannot correctly model the relation between number of blank answers and the grade point average. For the multiway ANOVA analysis the grade point average of students were therefore grouped into three bins containing an equal amount of participants ([16.7,66.9], [66.9,75.5],[75.5,88.6]) representing three levels of ability. When taking all the factors into account, the number of blank answers is significantly different across the grade point average groups (lower number of blanks for the higher grade point average group) and between the two scoring methods (fewer blanks with elimination testing with adapted scoring). The effect of the scoring method moreover interacts with grade point average: the effect of grade point average is larger for negative marking and almost non-existent with elimination testing with adapted scoring. These results confirm the third hypothesis: “elimination testing with adapted scoring reduces the number of blank answers in comparison with negative marking”.

**Table 11:** Multi-way ANOVA between number of blank answers and factors gender, master/scoring method, examination moment and binned grade point average.

	Paediatrics		Gynaecology	
response: number of blank answers	*F(2*,*659)*	*p*	*F(2*,*659)*	*p*
**examination moment (EM)**	3.628	0.057^ns^	0.235	0.628^ns^
**gender**	0.038	0.846^ns^	5.446	0.020*
**binned grade point average**	37.041	<0.001***	91.287	<0.001***
**master/scoring method**	150.683	<0.001***	218.196	<0.001***
**EM:gender**	0.397	0.529^ns^	0.272	0.601^ns^
**EM:GPA_bin**	0.086	0.918^ns^	0.689	0.502^ns^
**gender:GPA_bin**	0.142	0.868^ns^	0.468	0.626^ns^
**EM:SM**	0.176	0.675^ns^	0.009	0.925^ns^
**gender:SM**	1.502	0.221^ns^	5.759	0.017*
**GPA_bin:SM**	15.373	<0.001***	5.169	0.006**
**EM:gender:GPA_bin**	0.368	0.692^ns^	4.421	0.012*
**EM:gender:SM**	0.051	0.821^ns^	0.122	0.727^ns^
**EM:GPA_bin:SM**	0.220	0.803^ns^	0.042	0.960^ns^
**gender:GPA_bin:SM**	0.379	0.685^ns^	0.019	0.981^ns^
**EM:gender:GPA_bin:SM**	1,000	0.368^ns^	2.010	0.135^ns^

ANOVA Table for Type II tests. ANOVA model: number of blank answers ~ examination moment * gender * scoring method*GPA_bin. Degrees of freedom (numerator, denominator) for all factors: (1,659) except for those in combination with binned grade point average (2,659). GPA_bin = binned grade point average, SM = scoring method, EM = examination moment. Superscripts indicate levels of significance using the following coding

ns *p* > 0.05

* *p* < 0.05

** *p* < 0.01

*** *p* < 0.001.

Interestingly for Gynaecology there is a significant *impact of gender on the number of blanks* (female students have more blanks), which does not exist in Paediatrics. The Gynaecology gender-effect interacts with the scoring method: while the difference in blanks due to gender is large in negative marking, it is strongly reduced in elimination testing with adapted scoring. This supports the fourth hypothesis “elimination testing with adapted scoring reduces the gender bias in number of blank answers in comparison with negative marking”, at least for the course where a gender bias was found (Gynaecology).

#### Hypothesis 5: Reduction in guessing

In both elimination testing with adapted scoring and negative marking all students use at least once a *non-doubt answering pattern*. Elimination testing with adapted scoring however reduces the average percentage of questions with a non-doubt answering patterns in comparison with negative marking by 11.2% on average and by between 9.0% and 13.6%. Similarly, the two knowledge levels that could result from a non-doubt answering pattern (full knowledge and partial misconcept 1) are also lower in elimination testing with adapted scoring than in negative marking (S4 and S5 Tables). To test the statistical significance of these observations the multiple linear regression model developed to predict the score based on grade point average and other factors, was applied to predict the number of non-doubt answers. The results of the regression, shown in Table 12, indicate that the model explained 44% of the variance for Paediatrics (adjusted *R*^2^ = 0.444, residual standard error 3.044, *F*(10,672) = 55.41, *p <* 0.001) and 51% for Gynaecology (adjusted *R*^2^ = 0.512, residual standard error = 6.915, *F*(10,672) = 71.19, *p <* 0.001). The models first show that grade point average significantly predicts the number of non-doubt answers, where students with a higher grade point average have more non-doubt answers. The scoring method also significantly predicts the number of non-doubt answers for both exams with a large regression coefficient in each model. The interaction effect between grade point average and scoring method also predicts the number non-doubt answers: the grade point average effect is stronger in elimination testing with adapted scoring. Non-doubt answering patterns can be the result of students who believe to know the answer, or students who just pick one answer even when doubting between different alternatives. The latter behavior where students pick one alternative even when they are not entirely sure that this is the correct answer, is referred to as guessing. Therefore the observed reduction of non-doubt answering patters, even when corrected for grade point average, gender, and examination moment, provides indirect support for the fifth hypothesis: “elimination testing with adapted scoring reduces guessing in comparison with negative marking”.

**Table 12:** Results of multiple linear regression for predicting number of non-doubt answers.

	Paediatrics			Gynaecology		
	*β*	*t*(672)	*p*	*Β*	*t*(672)	*P*
Intercept	15.125 (2.221)	6.810	<0.001***	9.425 (4.257)	2.214	0.027*
grade point average	0.259 (0.031)	8.484	<0.001***	0.778 (0.061)	12.844	<0.001***
scoring method [negative marking]	15.110 (2.552)	5.922	<0.001***	37.936 (5.796)	6.545	<0.001***
gender [female]	-1.936 (1.880)	-1.030	0.303^ns^	-2.256 (4.118)	-0.548	0.584^ns^
examination moment [T2]	-0.799 (2.873)	-0.278	0.781^ns^	20.821 (5.306)	3.924	<0.001***
GPA*SM	-0.154 (0.035)	-4.351	<0.001***	-0.417 (0.080)	-5.187	<0.001***
GPA*gender	0.023 (0.026)	0.911	0.363^ns^	0.037 (0.059)	0.637	0.524^ns^
GPA*EM	0.004 (0.040)	0.100	0.920^ns^	-0.313 (0.077)	-4.077	<0.001***
GPA*SM*EM	-0.034 (0.476)	0.868	0.386^ns^	0.3478 (0.118)	2.956	0.003**
SM*gender	0.413 (0.052)	-0.651	0.515^ns^	-2.191 (1.082)	-2.026	0.043*

The table shows the regression coefficients (β) for both the courses (Paediatrics and Gynaecology) (N = 683, R^2^ = 0.444 for Paediatrics and 0.512 for Gynaecology). GPA = grade point average, SM = master/scoring method, EM = examination moment. Superscripts indicate levels of significance using the following coding

ns *p* > 0.05

* *p* < 0.05

** *p* < 0.01

*** *p* < 0.001.

For Gynaecology an interaction effect between gender and scoring method exists (male students had more non-doubt answers in negative marking than female students), but this difference is reversed in elimination testing with adapted scoring (female students have more non-doubt answers than male students).

### Questionnaire

#### Hypothesis 6: Test anxiety

Table 13 presents the results of the students’ responses on stress-related questions. Detailed results of the questionnaire can be found in the S2 Fig, S3 Fig, and S4 Fig. More than 60% of the students agree that the possibility to choose more than one answer in elimination testing with adapted scoring feels safe, while 44% of the students indicate that it felt unsafe. In negative marking, having to choose one answer feels risky for 63% of the students. For neither elimination testing with adapted scoring nor negative marking students indicate that the scoring method makes them feel more relaxed. Student report significantly higher stress levels with negative marking than with elimination testing with adapted scoring. However, when asked if negative marking causes more stress than elimination testing with adapted scoring, the average answer is not significantly different from neutral.

**Table 13:** Student responses on stress-related questions on elimination testing with adapted scoring and negative marking after receiving the exam score.

	question	N	mean	p-value t-test	mean male	mean female	p-value wilcoxon
**ETA**	I felt unsafe because I was able to choose more than one answer in elimination testing with adapted scoring.	180	2.83	0.058^ns^	2.47	3.15	<0.001***
	Being able to choose more than one answer in elimination testing with adapted scoring felt very safe.	183	3.45	<0.001***	3.58	3.34	0.13^ns^
	elimination testing with adapted scoring made me feel more relaxed, knowing that I can get a reasonable mark.	182	2.59	<0.001***	2.84	2.37	0.002**
	My stress levels were high with elimination testing with adapted scoring.	182	3.10	0.216^ns^	2.80	3.37	<0.001***
**NM**	Having to choose just one answer in negative marking feels very risky.	183	3.34	<0.001***	3.27	3.41	0.38^ns^
	Being able to choose just one answer in negative marking feels very safe.	181	2.98	0.787^ns^	2.99	2.97	0.91^ns^
	It makes me feel more relaxed, knowing that I can get a reasonable mark.	183	2.69	<0.001***	2.87	2.54	0.015*
	My stress levels were high with negative marking.	183	3.63	<0.001***	3.37	3.87	0.001**
	I would be more stressed with negative marking than with elimination testing with adapted scoring.	183	2.92	0.325^ns^	2.92	2.92	0.334^ns^

N indicates the number of responses. NM abbrebiates negative marking and ETA elimination testing with adapted scoring. The 5-point Likert scale of the questionnaire was converted to a numeric scale as follows: Strongly agree– 5, Agree– 4, Neither agree nor disagree– 3; Disagree– 2, Strongly disagree– 1. Superscripts indicate levels of significance using the following coding

ns *p* > 0.05

* *p* < 0.05

** *p* < 0.01

*** *p* < 0.001.

Regarding gender difference, female students agree more that choosing more than one answer in elimination testing with adapted scoring felt risky. Additionally male students report a significantly lower stress level than female students in both elimination testing with adapted scoring and negative marking. In negative marking male students agree significantly more than female students that they feel more relaxed knowing that they could get a reasonable mark, in elimination testing with adapted scoring this difference is not statistically significant.

#### Hypothesis 7: Preference

Table 14 presents the results of the students’ responses on questions comparing elimination testing with adapted scoring and negative marking. Full results of the questionnaire can be found in S2 Fig, S3 Fig and S4 Fig. Students find elimination testing with adapted scoring and negative marking equally difficult. Students do not agree that negative marking will lead to a higher score compared to elimination testing with adapted scoring, nor do they agree that elimination testing with adapted scoring will lead to a higher score compared to negative marking. When asked to compare their exam score for the exam with elimination testing with adapted scoring to their expectations, student indicated that they disagree that their expected score was higher than the actual score. Students strongly agree that elimination testing with adapted scoring requires more time. Students however also indicate they prefer elimination testing with adapted scoring rather than that they prefer negative marking, although this difference is not statistically significant.

**Table 14:** Student responses on comparative statements on elimination testing with adapted scoring and negative marking after receiving the exam score.

questions	N	mean	p-value t-test	mean male	mean female	p-value wilcoxon
Negative marking is more difficult than elimination testing with adapted scoring.	182	2.99	0.891^ns^	3.08	2.91	0.28^ns^
negative marking will lead to a higher score compared to elimination testing with adapted scoring.	175	2.80	0.009**	2.67	2.92	0.13^ns^
Negative marking will lead to a lower score compared to elimination testing with adapted scoring.	174	2.87	0.074^ns^	2.98	2.78	0.17^ns^
Elimination testing with adapted scoring will lead to a higher score compared to negative marking.	175	2.79	0.005**	2.93	2.66	0.081^ns^
There is a higher chance of getting answers right with elimination testing with adapted scoring than with negative marking.	182	3.62	<0.001***	3.62	3.62	0.84^ns^
I would be more stressed with negative marking than with elimination testing with adapted scoring.	183	2.92	0.325^ns^	2.92	2.92	0.97^ns^
After taking all aspects into consideration, I prefer negative marking.	183	2.85	0.109^ns^	2.77	2.93	0.34^ns^
After taking all aspects into consideration, I prefer elimination testing with adapted scoring.	183	3.16	0.074^ns^	3.26	3.08	0.28^ns^
I expected a higher mark for negative marking.	181	3.06	0.444^ns^	3.07	3.05	0.99^ns^
I expected a higher mark for elimination testing with adapted scoring.	181	2.71	<0.001***	2.62	2.79	0.27^ns^
I expected to do equally as well for both (elimination testing with adapted scoring or negative marking) tests.	179	2.80	0.008**	2.83	2.77	0.73^ns^
I prefer to be rewarded for knowing or guessing the answers exactly even though there is a penalty for answering or guessing incorrectly.	179	3.41	<0.001***	3.56	3.28	0.11^ns^
I prefer to be rewarded for demonstrating my partial and full knowledge rather than guessing what the right answer is.	182	3.84	<0.001***	3.72	3.95	0.24^ns^
I need more time to answer in elimination testing with adapted scoring compared to negative marking.	183	4.40	<0.001***	4.52	4.29	0.29^ns^

N indicates the number of responses. The 5-point Likert scale of the questionnaire was converted to a numeric scale as follows: Strongly agree– 5, Agree– 4, Neither agree nor disagree– 3; Disagree– 2, Strongly disagree– 1. Superscripts indicate levels of significance using the following coding

ns *p* > 0.05

* *p* < 0.05

** *p* < 0.01

*** *p* < 0.001.

No statistically significant differences between male and female students were found in any of the questions directly comparing elimination testing with adapted scoring and negative marking. Regarding instructions, 95% of the students agree that the instructions for elimination testing with adapted scoring were clear.

For the overarching question “After taking all aspects into consideration, I prefer negative marking” a regression analyses was used to investigate the influence of grade point average, exam score, course, gender, and examination moment. The results, detailed in S1 File, show that the preference of students is not influenced by the exam score, gender, and the examination moment. When taking into account grade point average the preference does depend on the course (and thus the master year) and the interaction between the course and grade point average. In detail: firstly, if elimination testing with adapted scoring was used for Paediatrics (2^nd^ master students), students prefer negative marking less than if elimination testing with adapted scoring was used for Gynaecology (1^st^ master students); secondly if elimination testing with adapted scoring was used for Paediatrics (1^st^ master students) students with higher grade point average have a higher preference for negative marking but if elimination testing with adapted scoring was used for Gynaecology (2^nd^ master students), there is no influence of grade point average on the preference.

## Discussion

This study comparing elimination testing with adapted scoring with negative marking was motivated by the search for an alternative for negative marking that still discourages guessing, but that is less disadvantageous for non-relevant student characteristics such as risk-aversion and that does not result in grade inflation. In the following, different aspects of the results are discussed in more detail. Afterwards the limitations of the present study are presented. Finally, overall conclusions are drawn.

### Grade inflation

Elimination testing with adapted scoring was selected in this study to alleviate the increase of the average score of the more traditional elimination testing scoring procedure in comparison with negative marking [22,28,31,32]. Prior theoretical analysis using prospect theory, which is a model for decision making under uncertainty, predicted that elimination testing with adapted scoring would not suffer from this grade inflation in comparison with negative marking [32]. This prior work used prospect theory to calculate the answering patterns for students with different abilities and risk aversion, and from these answering patterns the expected scores. This study empirically confirms this prediction: in contrast with grade point average and gender, the scoring method does not significantly influence the exam score after correcting for the background variables (examination moment, gender and ability). In accordance with the empirical results, students disagree in the questionnaire with the statement that either negative marking or elimination testing with adapted scoring leads to a higher score.

The absence of a significant difference between average exam score between elimination testing with adapted scoring and negative marking does not mean that the score of particular students would not change between elimination testing with adapted scoring and negative marking. As an example: the average grade of risk-averse student may increase, while that of risk-seeking students may decrease. Therefore, future research regarding the impact of elimination testing with adapted scoring on particular subgroups of students is needed.

### Answering patterns–partial knowledge

The ability to express partial knowledge is an interesting feature of elimination testing with adapted scoring. Similar as for elimination testing with adapted scoring in this study, Bradbard et al. [31] found that a scoring method equivalent to elimination testing with traditional scoring allows to measure partial knowledge. During discussions, students appreciated that elimination testing with adapted scoring offered the possibility to show their partial knowledge as it allows for a more faithful representation of their actual knowledge. After receiving their grades, students confirmed in the questionnaire that the possibility of elimination testing with adapted scoring to choose more than one answer feels safe. However, both students and teachers indicated that receiving partial knowledge points while not eliminating a “dangerously wrong” alternative, is contested, especially in medicine. This comment contrasts with recommendations found in literature [28,31] that support the use of elimination testing specifically in content areas where partial or full misinformation results in life-threatening consequences. This concern is partially alleviated by the fact that the maximum score for partial knowledge in elimination testing with adapted scoring is always lower than ½: as a result students can never pass the exam by only showing partial knowledge. This is in contrast with the most common scoring method for elimination testing, which rewards partial knowledge more generously (up to ¾ for 5 alternatives).

This study confirms earlier findings regarding elimination testing with adapted scoring in first-year engineering exams with multiple-choice questions [33]: the vast majority of students actually use elimination testing with adapted scoring to express their partial knowledge by using answering patterns expressing doubt. Even more, despite the difference in the students’ program (engineering science versus medicine) and the students’ experience (freshman bachelor without experience in multiple-choice questions versus master students with prior experience), the average percentage of answers where students express doubt is similar (between 10.7% and 22% in the engineering exams versus between 14.7% and 18.7% for the different courses and examination moments in the medicine exams). However, differences are observed for the percentage of students using doubt on at least one question: while the percentages in this study (between 95.5% and 98.4% for the different courses and examination moments) are very similar to the Philosophy exam in [33] (96.7%), they are higher than the engineering exam on Electrical Circuits in [33] (79.4%). Further research is needed to explain this difference.

### Risk-aversion and guessing

According to [9, 10], the penalty of negative marking for wrong answers leads to different answering patterns and scores of students with equal ability but different risk-aversion. In particular, risk-averse students with partial knowledge would rather leave a question blank than to guess an answer. Furthermore, as risk-aversion is claimed to be higher in female students, female students may be disadvantaged by negative marking [17,18]. The present study looked at the answering patterns of blanks and non-doubts to study the effect of the scoring method on guessing.

This study found that elimination testing with adapted scoring reduces the number of *blank answers* in comparison with negative marking. Moreover, elimination testing with adapted scoring significantly reduced the difference in blank answer between male and female students in an exam where a gender difference under negative marking was observed. With respect to an earlier result with elimination testing with adapted scoring in first-year engineering exams with multiple-choice questions [33], the percentage of students leaving at least one question blank and the average percentage of questions left blank per student reported in this study is similar to the engineering exam of Philosophy (56.6% of students, 4.9% of questions) but lower than the engineering exam of the technical course Electrical Circuits (88.1% of students, 19.7% of questions). Obviously student experience, the exam subject, and difficulty impact the number of blank answers.

This study found that, in comparison with negative marking, elimination testing with adapted scoring significantly reduced the number of *non-doubt answering patterns*, i.e. answers where students eliminate all but one alternative. This indirectly supports the hypothesis that elimination testing with adapted scoring reduces guessing in comparison with negative marking. Similarly, Bradbard et al. [31] found that an elimination procedure with a scoring scheme equivalent to elimination testing with traditional scoring reduces guessing. Our finding partially refutes the criticism of Lesage et al. [5] concerning elimination testing, in which they state that elimination testing misses the purpose of countering guessing behaviour as students can still guess in an attempt to increase their score.

Summarizing, this study shows that elimination testing with adapted scoring results in a shift from non-doubt and blank answers in negative marking to answering patterns showing doubt in elimination testing with adapted scoring. One possible interpretation is that, in line with the aim of using elimination testing with adapted scoring, this shift is caused by a reduction of guessing in case the student only has partial knowledge of the item. However, another interpretation could be that elimination testing with adapted scoring drives risk-averse students to answering patterns expressing doubt rather than picking a single answer, even when they are quite sure about their answers. As such, elimination testing with adapted scoring could still induce differences between students based on their risk-aversion. Since there is no paired data in this study to compare the answers of an individual student on a specific question with both scoring methods, it is not possible to unequivocally confirm one interpretation. However, there is indirect support for the first ‘guessing-is-reduced’ interpretation. Similarly, this study can’t draw conclusions regarding “partial guessing” behaviour that occurs when e.g. a student doubts between three alternatives but decides to only keep two of these in the answering pattern.

This study found an interesting interaction effect between gender and the scoring method for the number of non-doubt answers in Gynaecology. Male students had more non-doubt answers (both correct and wrong answers) in negative marking than female students, but female students have more non-doubt answers than male students in elimination testing with adapted scoring. Assuming that female students guess less (more risk-averse) and are better at Gynaecology, as confirmed by the results, their number of non-doubt answers would not change between negative marking and elimination testing with adapted scoring, while male students may guess less given the opportunity to express their doubt and get a statistical equivalent reward in elimination testing with adapted scoring, thus reducing their number of non-doubt answers.

When using answering patterns expressing doubt in elimination testing with adapted scoring, this paper showed that students most often doubt between two alternatives. Interestingly, this is also the situation in which the difference between negative marking and elimination testing with adapted scoring is the largest. In negative marking students then have to decide to either “guess” between the two remaining options (resulting in a score of +1 or -1/4) or leave the question blank (resulting in a score of 0). The fear for the penalty creates a difference based on the risk-aversion of students. In elimination testing with adapted scoring however, students can by showing their doubt get the “expected score” of 3/8, an additional safe answering pattern besides the ones available in negative marking.

### Anxiety

Similar to [33], students report lower stress levels in elimination testing with adapted scoring compared to negative marking in a questionnaire after receiving their exam scores. Regarding gender, male students report lower stress levels than female students in both elimination testing with adapted scoring and negative marking, which is in accordance with the significantly higher stress levels reported by female medical students than by male students [24].

### Preference

Bond et al. compared student experiences and opinions using a similar questionnaire in a formative test where students replied each of the 25 multiple-choice questions using both negative marking and elimination testing with traditional scoring [22]. Despite the lower scores for partial knowledge of elimination testing with adapted scoring in this paper in comparison with elimination testing with traditional scoring of [22], students responses on questions comparing methods are similar to [22]. Students similarly reported higher stress levels in negative marking than in elimination testing with adapted scoring, disagreed that negative marking will lead to a higher score compared to elimination testing with adapted scoring, reacted neutrally to the reverse statement that ET will lead to a higher score compared to negative marking, and agreed that there is a higher chance of getting answers right with elimination testing with adapted scoring than with negative marking. Similar to the Level_2 students but opposed to the Level_1 students of [22], the 1^st^ and 2^nd^ master students in this study preferred elimination testing with adapted scoring over negative marking. In line with the conclusions of [22] this paper found that 2^nd^ master students prefer elimination testing with adapted scoring more than 1^st^ master students, although this effect could also be attributed to the particular course due to the crossed test design. The preference of elimination testing with adapted scoring over negative marking is in line with the favorable reception of students when the elimination testing with adapted scoring method was originally introduced [34], and the evaluation of elimination testing with adapted scoring in first-year engineering exams [33].

### Time

Elimination testing was shown to require additional time [8]. Similar to the freshman engineering students in [33], students in this study indicated that elimination testing with adapted scoring requires extra time in comparison with negative marking. Students complain about the extra time needed to color all the “circles” on the answer sheet, even if they know the correct answer (remind that in elimination testing students have to indicate all alternatives they believe to be incorrect). Students found this particularly important for the Gynaecology exam with 80 questions and 5 alternatives. Further research is needed to study the main factors for the additional time needed. Is it mainly to the coloring of the extra circles, or also due to the extra answering patterns offered by elimination testing?

### Exclusion versus inclusion-scoring

Two main approaches for indicating partial knowledge, without including explicit confidence levels or alternative weighting, exist: elimination testing (exclusion-scoring) and subset selection (inclusion-scoring [3] or liberal multiple-choice [29]). In contrast with elimination, students indicate in subset selection the answers they want to include. While both methods can be conceived such that scoring is identical, and are just the “complement” of each other, Bereby-Meyer et al. showed that students will act differently: students will take greater risks in inclusion scoring than in exclusion scoring [36]. Even more, Jaradat and Tollefson [37] found that scores of a test administered with both inclusion and exclusion were not as strongly correlated as expected. In the current study, the scoring method was explained to students as elimination testing (i.e. reward for eliminated distractors, penalty for eliminated correct answer). The answer sheets however contained two rows (Fig 1): in the top row (“cannot be”) students mark the alternatives they eliminate, in the bottom row (“could be”) students mark the alternatives they believe to be correct. While this design was made because of practical reasons, the bottom row exposed the students to an inclusion test strategy. Interestingly, after the exam some students requested to only keep the second row (inclusion), rather than the top row (exclusion). This exposes an opportunity for future work, where elimination testing with adapted scoring [34] is reformulated for inclusion scoring or subset selection.

### Scoring method complexity

Elimination testing is criticized for its complexity of scoring and instructions. Students requested an additional information session and practicing modules to introduce elimination testing with adapted scoring. After the exam, 95% of the students agreed that the instructions were clear. In the questionnaire students confirmed that the extra difficulty of elimination testing with adapted scoring can be easily addressed by appropriate instructions. This is in line with the findings of Bradbard et al. [31] but in contrast with the finding of Jaradat and Tollefson [37], where students found the test directions confusing despite prior practice. This paper focused in experienced 1^st^ and 2^nd^ year master students, so no conclusion concerning more naive test makers can be made. Similar positive experiences have however been reported for bachelor students [22,32,33] and college students [31].

### Limitations and future work

This study comparing elimination testing with adapted scoring and negative marking was done in a particular setting: two courses of the master of Medicine of a large Belgian university. The students involved in this study were therefore, well-experienced 1^st^ and 2^nd^ master students. Earlier research has indicated that students might react differently on scoring methods depending on their experience level [22].

Both exams in this study used five alternatives, while this is criticized in literature [38–42] as it was found that questions seldom contain more than two useful distractors. These studies suggest that using three alternatives would maximize efficiency whilst maintaining, or possibly improving, psychometric quality, through allowing a greater number of questions per exam. The two courses in our study are typically well-mastered by students, resulting in high average scores, high percentages of full knowledge answers, and limited amount of blanks. The real-life exam setting prevented that students would simultaneously use two scoring procedures for the same exam. As such, no within-subject comparison between elimination testing with adapted scoring an negative marking could be achieved. The resulting crossed test design required a more advanced statistical analysis and careful interpretation of results. In particular, the two courses Paediatrics and Gynaecology had to be analyzed separately such that if the analysis of both courses are consistent, the effects can be attributed to the scoring method and not to the master level. However, when interpreting the course by course analyses, care had to be taken as the factor scoring method both encompasses master level and scoring method.

This study interprets differences in answering patterns and scores between men and women in light of risk-aversion, while other factors have been shown to induce gender differences (e.g., instructions [17], test preparation [23], test anxiety [24], subject area [6], extrinsic rewards or stakes in general [19,25], question difficulty [6,19], and stereotype threat [19]). Moreover, only differences between male and female students have been studied while differences between other student groups, such as students with disabilities are also of interest [43]. Additionally, this paper focused on studying the container of “doubt” answering pattern while future research can dig deeper into the different answering patterns (doubt between two, three, and four alternatives).

As a measure for ability, this study uses grade point average. grade point average is a measure for academic achievement, which is strongly correlated with general mental ability, but is also affected by other factors such as motivation. Bacon and Bean showed that grade point average often correlates highly with variables of interest to educational researchers and can therefore be used to increase the statistical power of their research studies [35].

Beside the scoring method, instructions for this scoring method have been shown to influence gender-related differences in tests with multiple-choice questions [17]. Additionally, ‘framing’ in multiple-choice exams, i.e. how students “perceive themselves as a function of their current situation, abilities, previous achievements, and aspirations” [3] and perception of the test situation and of the consequences of guessing affects student behaviour [3]. Future work should therefore dig deeper on the design of instructions for elimination testing with adapted scoring that might further reduce gender-induced differences.

Future work should furthermore include a reliability study of elimination testing with adapted scoring, including a comparison with negative marking and the impact of the number of alternatives. Burton [2] shows that guessing, the number of questions, and the number of alternatives affect test reliability. In [9] Burton postulates that negative marking can improve test reliability by penalizing misinformation as well as by discouraging guessing. As elimination testing with adapted scoring penalizes misinformation, and even reduces guessing with negative marking, the test reliability is expected to increase. On the other hand, students indicate they need more time for elimination-type multiple-choice question [8], which might lead to a reduction of the total number of questions in order to guard total test time. As a decrease in the total number of questions negatively affects test reliability, the increased required time of elimination testing with adapted scoring is of concern. Earlier studies of the reliability of elimination testing with traditional scoring showed mixed results: Hakstian and Kansup [8] and Jaradat and Tollefson [37] report little, if any, improvement of reliability with respect to traditional scoring methods while Ben-Simon et al. [26] and Bradbard et al. [31] reported more optimal reliability with respect to negative marking.

The stress induced by the scoring methods was assessed using a post-test questionnaire, after students received their grades. The reuse of the questionnaire of Bond et al. [22] allowed for comparison with this prior work comparing elimination testing with traditional scoring and negative marking. This questionnaire is however not a validated instrument to assess test anxiety, and therefore results should be interpreted with care. Future work should consider validated psychometric instruments to assess task-induced stress, such as the Dundee stress state questionnaire [44,45]. This is particularly interesting as the different stress factors measured using the questionnaire have shown to impact performance.

Finally, this study focused on comparing elimination testing with adapted scoring and negative marking, without considering other scoring methods. Regarding the promising results of elimination testing with adapted scoring, comparison with other scoring methods should be subject of future research.

## Conclusion

This study shows that elimination testing using the scoring introduced by Arnold and Arnold [34] (elimination testing with adapted scoring) is a valuable alternative for negative marking when looking for a scoring method that discourages guessing. In contrast to traditional scoring of elimination testing, elimination testing with adapted scoring does not result in grade inflation in comparison with negative marking. This study shows that elimination testing with adapted scoring reduces blank answers and finds strong indications for the reduction of guessing in comparison with negative marking. Finally, students prefer elimination testing with adapted scoring over negative marking and report lower stress levels in elimination testing with adapted scoring in comparison with negative marking.

## Supporting information

S1 Fig Relation between GPA and exam score. The graph shows the strong and approximately linear relation between GPA (%) and exam score.
(PDF)

S2 Fig Survey responses for questions regarding elimination testing with adapted scoring. (PDF)

S3 Fig Survey responses for questions regarding negative marking (NM). (PDF)

S4 Fig Survey responses for questions comparing negative marking (NM) and elimination testing with adapting scoring (ETA). (PDF)

S1 Table Survey statements. ETA abbreviates elimination testing with adapted scoring.
(PDF)

S2 Table Percentage of students that show different answering patterns on at least one multiple choice question. The answering patterns are defined in Table 3. Doubt is an aggregation of doubt two, doubt three, and doubt four. NM = negative marking, ETA = elimination testing with adapted scoring, T1 = exam moment 1, T2 = exam moment 2
(PDF)

S3 Table Answering patterns: average number of questions per student in a multiple choice question. Average number of multiple choice questions per student and standard deviation (between brackets) showing different answering patterns. Pediatrics exams have 40 questions and Gynaecology exams have 80 questions. The answering patterns are defined in Table 3. Doubt is an aggregation of doubt two, doubt three, and doubt four.
(PDF)

S4 Table Percentage of students that show the different knowledge levels on at least one question in a multiple choice exam. The knowledge levels are defined in Table 3. NM abbreviates negative marking. ETA abbreviates elimination testing with negative marking.
(PDF)

S5 Table Average number of questions in a particular knowledge level per student in a multiple choice exam. The knowledge levels are defined in Table 3. Partial knowledge (PK) is an aggregation of partial knowledge 1 (PK1), partial knowledge 2 (PK2), and partial knowledge 3 (PK3). PMI is an aggregation of partial misconcept 2 (PM2), partial misconcept 3 (PM3), and total misconcept (TM). Misconcept (M) is an aggregation of partial misconcept 1 (PM1), partial misconcept 2 (PM2), partial misconcept 3 (PM3), and total misconcept (TM). M = male, F = female.
(PDF)

S1 File Preference questionnaire response analyses. (PDF)
