# Supplementary material for: Elimination testing with adapted scoring reduces guessing and anxiety in multiple-choice assessments, but does not increase grade average in comparison with negative marking
Source: PLoS One. 2018 Oct 2;13(10):e0203931. doi: 10.1371/journal.pone.0203931 (PMC6168139; doi:10.1371/journal.pone.0203931)

S2 Fig. Relation between GPA and exam score. The graph shows the strong and approximately linear relation between GPA (%) and exam score.

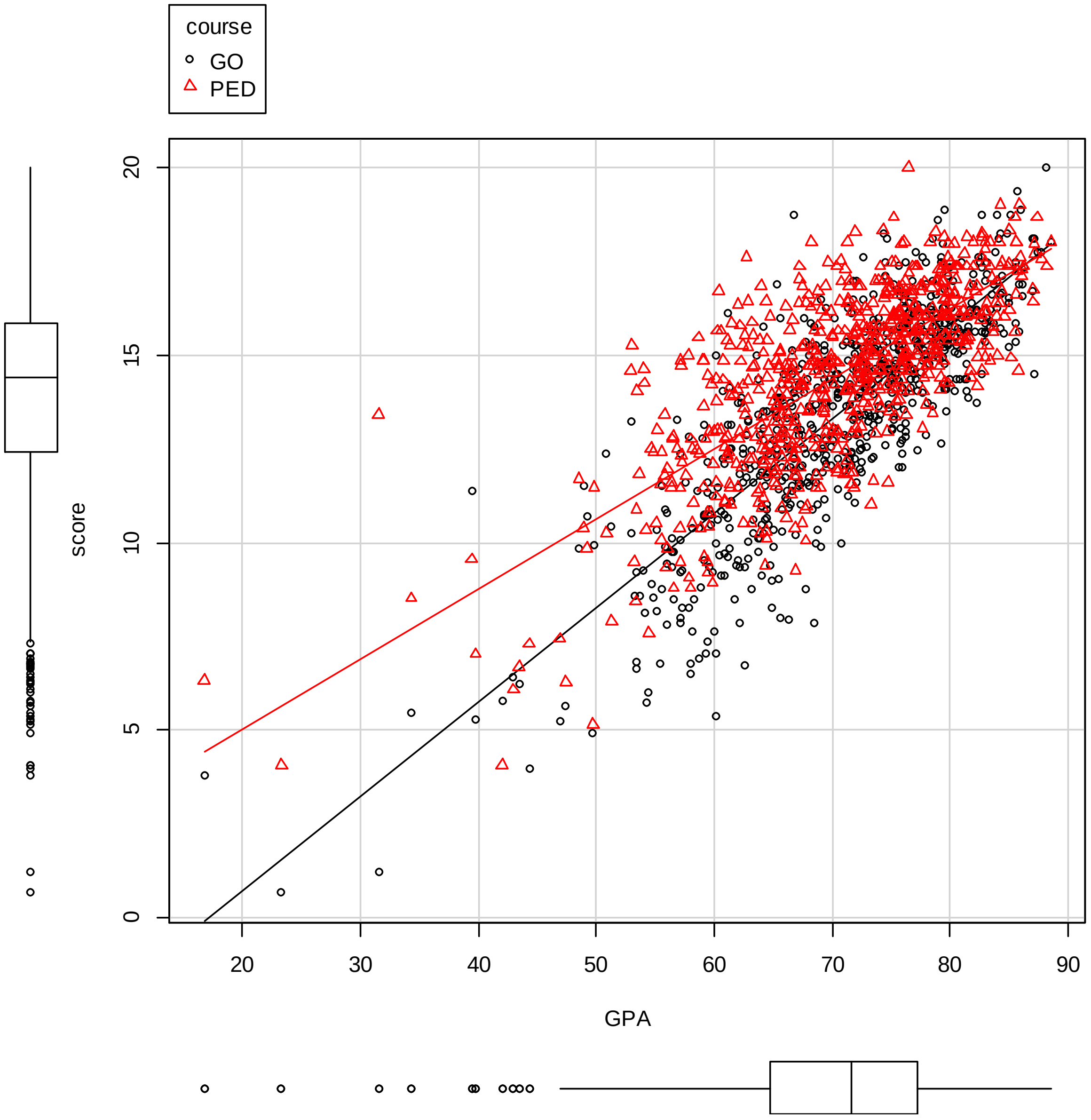

Supplement: S1 Fig — The graph shows the strong and approximately linear relation between GPA (%) and exam score. (PDF) [file pone.0203931.s001.pdf]
