# Supplementary figures and images for: Elimination testing with adapted scoring reduces guessing and anxiety in multiple-choice assessments, but does not increase grade average in comparison with negative marking
Source: PLoS One. 2018 Oct 2;13(10):e0203931. doi: 10.1371/journal.pone.0203931 (PMC6168139; doi:10.1371/journal.pone.0203931)

**S7 Fig. Survey responses for questions regarding elimination testing with adapted scoring (ETA).**

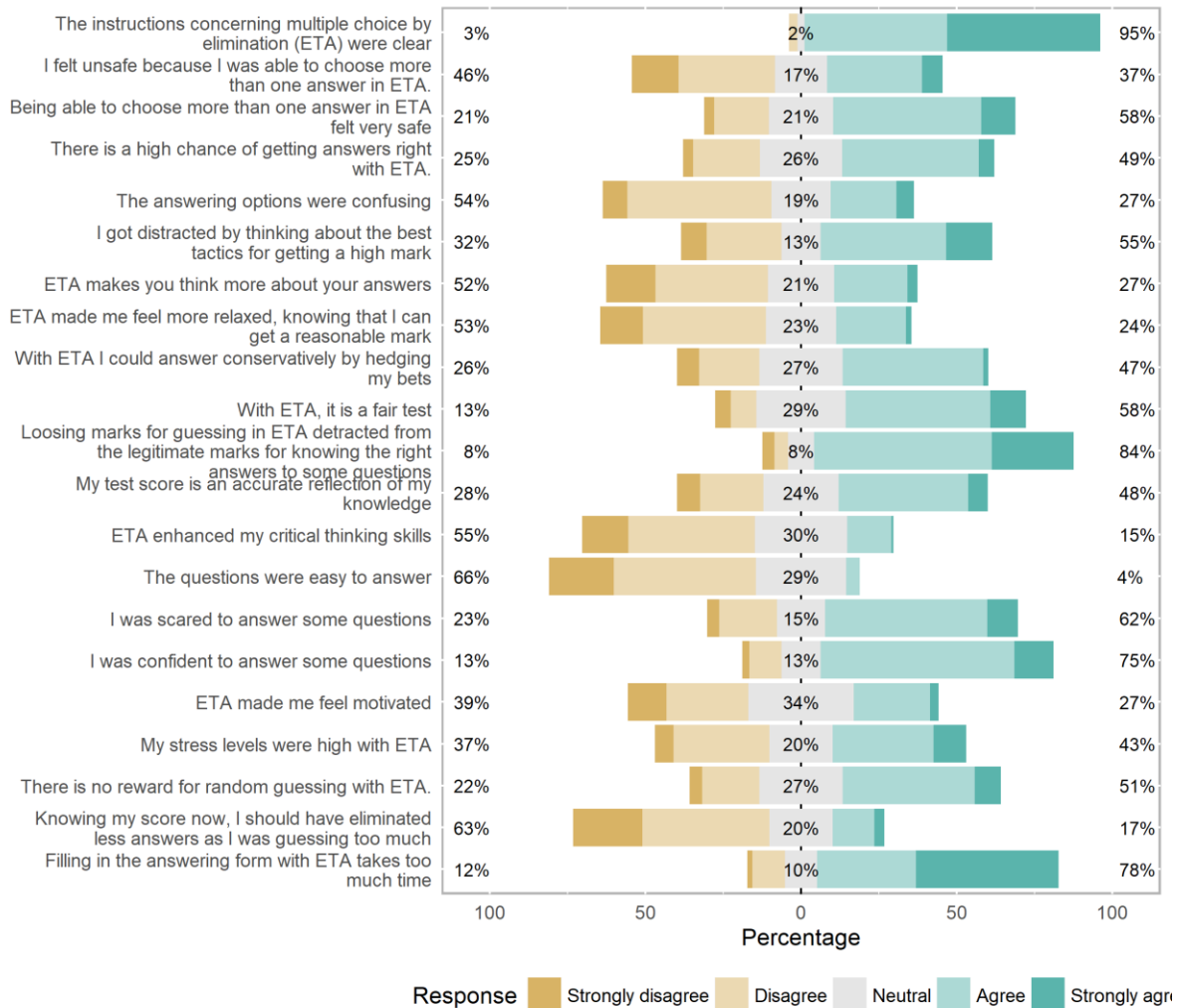

Supplement: S2 Fig — (PDF) [file pone.0203931.s002.pdf]

S8 Fig. Survey responses for questions regarding negative marking (NM).

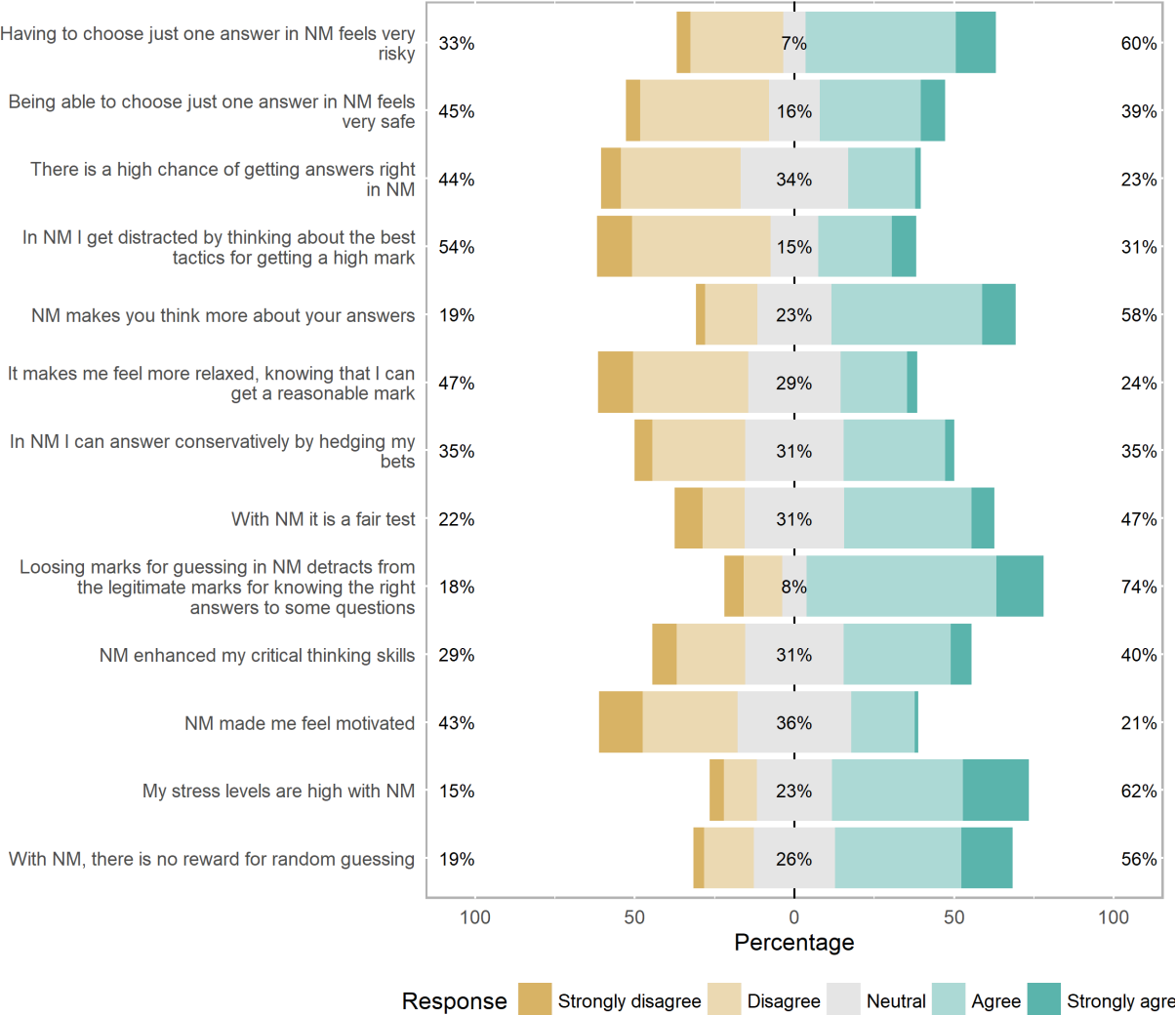

Supplement: S3 Fig — (PDF) [file pone.0203931.s003.pdf]
