# Supplementary material for: Elimination testing with adapted scoring reduces guessing and anxiety in multiple-choice assessments, but does not increase grade average in comparison with negative marking
Source: PLoS One. 2018 Oct 2;13(10):e0203931. doi: 10.1371/journal.pone.0203931 (PMC6168139; doi:10.1371/journal.pone.0203931)

S9 Fig. Survey responses for questions comparing negative marking (NM) and elimination testing with adapting scoring (ETA).

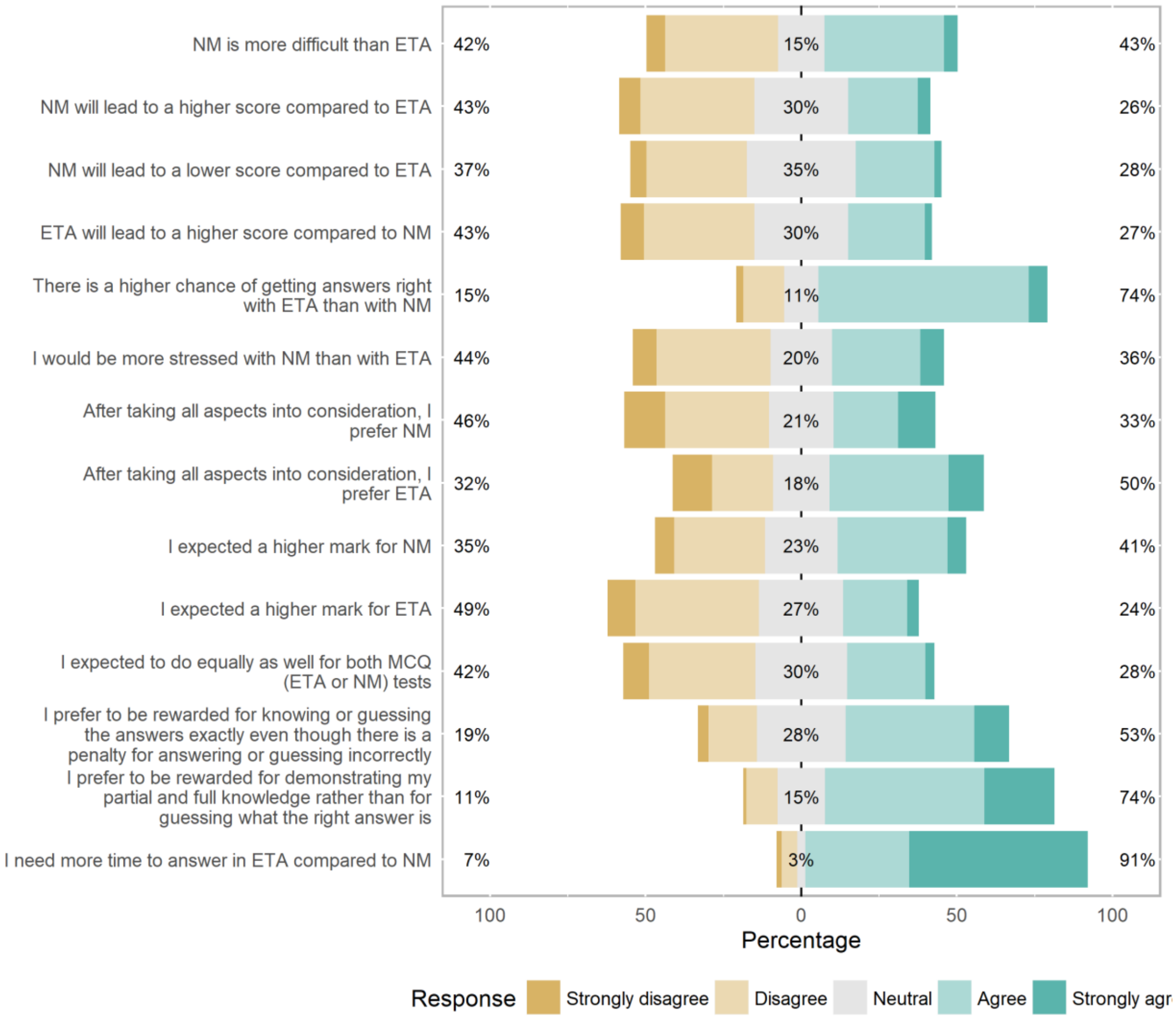

Supplement: S4 Fig — (PDF) [file pone.0203931.s004.pdf]
