# Supplementary material for: Elimination testing with adapted scoring reduces guessing and anxiety in multiple-choice assessments, but does not increase grade average in comparison with negative marking
Source: PLoS One. 2018 Oct 2;13(10):e0203931. doi: 10.1371/journal.pone.0203931 (PMC6168139; doi:10.1371/journal.pone.0203931)
Supplement: S1 Table — ETA abbreviates elimination testing with adapted scoring. (PDF) [file pone.0203931.s005.pdf]

S1 Table. Survey statements. ETA abbreviates elimination testing with adapted scoring. NM abbreviates negative marking.

subject	statement
**ETA**	The instructions concerning multiple choice by elimination (ETA) were clear.
	The instructions concerning multiple choice by elimination (ETA) were clear.
	I felt unsafe because I was able to choose more than one answer in ETA.
	Being able to choose more than one answer in ETA felt very safe.
	There is a high chance of getting answers right with ETA.
	The answering options were confusing.
	I got distracted by thinking about the best tactics for getting a high mark.
	ETA makes you think more about your answers.
	ETA made me feel more relaxed, knowing that I can get a reasonable mark.
	With ETA I could answer conservatively by hedging my bets.
	With ETA, it is a fair test.
	Loosing marks for guessing in ETA detracted from the legitimate marks for knowing the right answers to some questions.
	My test score is an accurate reflection of my knowledge.
	ETA enhanced my critical thinking skills.
	The questions were easy to answer.
	I was scared to answer some questions.
	I was confident to answer some questions.
	ETA made me feel motivated.
	My stress levels were high with ETA.
	There is no reward for random guessing with ETA.
	Knowing my score now, I should have eliminated less answers as I was guessing too much.
	Filling in the answering form with ETA takes too much time.
**NM**	Having to choose just one answer in NM feels very risky.
	Being able to choose just one answer in NM feels very safe.
	There is a high chance of getting answers right in NM.
	In NM I get distracted by thinking about the best tactics for getting a high mark.
	NM makes you think more about your answers.
	It makes me feel more relaxed, knowing that I can get a reasonable mark.
	In NM I can answer conservatively by hedging my bets.
	With NM it is a fair test.
	Loosing marks for guessing in NM detracts from the legitimate marks for knowing the right answers to some questions.
	NM enhanced my critical thinking skills.
	NM made me feel motivated.
	My stress levels are high with NM.
	With NM, there is no reward for random guessing.
**ETA vs NM**	NM is more difficult than ETA.
	NM will lead to a higher score compared to ETA.
	NM will lead to a lower score compared to ETA.
	ETA will lead to a higher score compared to NM.
	There is a higher chance of getting answers right with ETA than with NM.
	I would be more stressed with NM than with ETA.
	After taking all aspects into consideration, I prefer NM.
	After taking all aspects into consideration, I prefer ETA.
	I expected a higher mark for NM.
	I expected a higher mark for ETA.
	I expected to do equally as well for both MCQ (ETA or NM) tests.
	I prefer to be rewarded for knowing or guessing the answers exactly even though there is a penalty for answering or guessing incorrectly.
	I prefer to be rewarded for demonstrating my partial and full knowledge rather than for guessing what the right answer is.
	I need more time to answer in ETA compared to NM.
