# Supplementary material for: Elimination testing with adapted scoring reduces guessing and anxiety in multiple-choice assessments, but does not increase grade average in comparison with negative marking
Source: PLoS One. 2018 Oct 2;13(10):e0203931. doi: 10.1371/journal.pone.0203931 (PMC6168139; doi:10.1371/journal.pone.0203931)
Supplement: S2 Table — The answering patterns are defined in Table 3. Doubt is an aggregation of doubt two, doubt three, and doubt four. NM = negative marking, ETA = elimination testing with adapted scoring, T1 = exam moment 1, T2 = exam moment 2 (PDF) [file pone.0203931.s006.pdf]

S3 Table. Percentage of students that show different answering patterns on at least multiple choice question. The answering patterns are defined in Table 3. Doubt is an aggregation of doubt two, doubt three, and doubt four.

		Pediatrics				Gynaecology			
		T1		T2		T1		T2	
		NM	ETA	NM	ETA	NM	ETA	NM	ETA
no doubt	tot	100	100	100	100	100	100	100	100
	male	100	100	100	100	100	100	100	100
	female	100	100	100	100	100	100	100	100
doubt two	tot	-	90.5	-	95.5	-	91.7	-	97.5
	male	-	93.7	-	94.4	-	89.9	-	94.7
	female	-	87.6	-	96.7	-	93.0	-	100
doubt three	tot	-	61.9	-	59.2	-	55.3	-	82.4
	male	-	51.9	-	56.2	-	59.6	-	78.9
	female	-	70.8	-	62.2	-	52.3	-	85.5
doubt four	tot	-	30.4	-	29.1	-	49.8	-	54.6
	male	-	24.1	-	27.0	-	49.4	-	54.4
	female	-	36.0	-	31.1	-	50.0	-	54.8
blank	tot	70.0	29.8	80.7	49.7	92.3	65.4	91.6	77.3
	male	71.9	24.1	84.2	52.8	87.3	60.7	88.8	73.7
	female	68.8	34.8	77.4	46.7	96.6	68.8	94.4	80.6
doubt	tot	-	95.8	-	97.2	-	96.8	-	98.3
	male	-	96.2	-	95.5	-	95.5	-	96.5
	female	-	95.5	-	98.9	-	97.7	-	100

NM = negative marking, ETA = elimination testing with adapted scoring, T1 = exam moment 1, T2 = exam moment 2
