# Supplementary material for: Elimination testing with adapted scoring reduces guessing and anxiety in multiple-choice assessments, but does not increase grade average in comparison with negative marking
Source: PLoS One. 2018 Oct 2;13(10):e0203931. doi: 10.1371/journal.pone.0203931 (PMC6168139; doi:10.1371/journal.pone.0203931)
Supplement: S3 Table — Average number of multiple choice questions per student and standard deviation (between brackets) showing different answering patterns. Pediatrics exams have 40 questions and Gynaecology exams have 80 questions. The answering patterns are defined in Table 3. Doubt is an aggregation of doubt two, doubt three, and doubt four. (PDF) [file pone.0203931.s007.pdf]

S4 Table. Average number of questions for different answering patterns per student in a multiple choice question. Average number of multiple choice questions per student and standard deviation (between brackets) showing different answering patterns. Pediatrics exams have 40 questions and Gynaecology exams have 80 questions. The answering patterns are defined in Table 3. Doubt is an aggregation of doubt two, doubt three, and doubt four.

		**Pediatrics**				**Gynaecology**			
		**T1**		**T2**		**T1**		**T2**	
		**NM**	**ETA**	**NM**	**ETA**	**NM**	**ETA**	**NM**	**ETA**
**no doubt**	**tot**	37.75(2.36)	33.69(4.30)	37.11(2.68)	33.01(4.20)	72.45(5.88)	64.51(11.11)	72.05(6.44)	61.45(10.11)
	**male**	37.60(2.41)	33.62(4.68)	37.19(2.58)	33.12(4.42)	73.41(5.68)	63.73(11.66)	72.63(6.12)	60.16(10.64)
	**female**	37.86(2.32)	33.75(3.93)	37.03(2.78)	32.90(3.97)	71.60(5.92)	65.05(10.68)	71.48(6.70)	62.65(9.44)
**doubt two**	**tot**	-	4.11(3.23)	-	4.47(2.86)	-	4.45(4.21)	-	10.29(5.95)
	**male**	-	4.59(3.74)	-	4.67(3.25)	-	4.61(4.62)	-	11.07(6.87)
	**female**	-	3.69(2.62)	-	4.28(2.41)	-	4.34(3.90)	-	9.58(4.85)
**doubt three**	**tot**	-	1.18(1.23)	-	1.34(1.55)	-	1.35(1.99)	-	3.03(2.76)
	**male**	-	1.04(1.34)	-	1.11(1.31)	-	1.47(2.01)	-	3.11(2.97)
	**female**	-	1.30(1.12)	-	1.56(1.72)	-	1.26(1.97)	-	2.97(2.55)
**doubt four**	**tot**	-	0.45(0.80)	-	0.37(0.68)	-	1.08(1.58)	-	1.47(2.11)
	**male**	-	0.33(0.67)	-	0.31(0.61)	-	1.12(1.63)	-	1.42(2.03)
	**female**	-	0.55(0.89)	-	0.43(0.73)	-	1.05(1.54)	-	1.52(2.18)
**blank**	**tot**	2.25(2.36)	0.57(1.09)	3.05(2.74)	0.80(1.08)	7.55(5.88)	2.65(3.52)	7.95(6.44)	3.75(4.91)
	**male**	2.40(2.41)	0.42(0.89)	3.28(2.73)	0.78(0.96)	6.59(5.68)	2.81(3.67)	7.37(6.12)	4.25(4.90)
	**female**	2.14(2.32)	0.71(1.23)	2.84(2.74)	0.83(1.19)	8.40(5.92)	2.55(3.41)	8.52(6.70)	3.29(4.87)
**doubt**	**tot**	-	5.74(4.00)	-	6.18(3.88)	-	12.84(9.41)	-	14.80(8.07)
	**male**	-	5.96(4.50)	-	6.10(4.20)	-	13.46(9.96)	-	15.60(8.99)
	**female**	-	5.54(3.49)	-	6.27(3.53)	-	12.41(8.97)	-	14.06(7.04)
