# Supplementary material for: Elimination testing with adapted scoring reduces guessing and anxiety in multiple-choice assessments, but does not increase grade average in comparison with negative marking
Source: PLoS One. 2018 Oct 2;13(10):e0203931. doi: 10.1371/journal.pone.0203931 (PMC6168139; doi:10.1371/journal.pone.0203931)
Supplement: S4 Table — The knowledge levels are defined in Table 3. NM abbreviates negative marking. ETA abbreviates elimination testing with negative marking. (PDF) [file pone.0203931.s008.pdf]

S5 Table. Percentage of students that show the different knowledge levels on at least one question in a multiple choice exam. The knowledge levels are defined in Table 3. NM abbreviates negative marking. ETA abbreviates elimination testing with negative marking.

		Pediatrics				Gynaecology			
		T1		T2		T1		T2	
		NM	ETA	NM	ETA	NM	ETA	NM	ETA
Full Knowledge	tot	100	100	100	100	100	100	100	100
	male	100	100	100	100	100	100	100	100
	female	100	100	100	100	100	100	100	100
Partial Knowledge 1	tot	-	88.7	-	94.4	-	94.0	-	97.5
	male	-	89.9	-	92.1	-	94.4	-	94.7
	female	-	87.6	-	96.7	-	93.8	-	100
Partial Knowledge 2	tot	-	57.1	-	58.1	-	77.0	-	79.8
	male	-	49.4	-	56.2	-	78.7	-	73.7
	female	-	64.0	-	60.0	-	75.8	-	85.5
Partial Knowledge 3	tot	-	28.0	-	29.1	-	47.0	-	53.8
	male	-	20.3	-	27.0	-	48.3	-	52.6
	female	-	34.8	-	31.1	-	46.1	-	54.8
No Knowledge	tot	70.0	29.8	80.7	49.7	92.3	65.4	91.6	77.3
	male	71.9	24.1	84.2	52.8	87.3	60.7	88.8	73.7
	female	68.8	34.8	77.4	46.7	96.6	68.8	94.4	80.6
Partial Misconcept 1	tot	100	99.4	100	100	100	100	100	100
	male	100	98.7	100	100	100	100	100	100
	female	100	100	100	100	100	100	100	100
Partial Misconcept 2	tot	-	51.2	-	63.1	-	76.0	-	79.0
	male	-	57.0	-	60.7	-	76.4	-	80.7
	female	-	46.1	-	65.6	-	75.8	-	77.4
Partial Misconcept 3	tot	-	12.5	-	14.0	-	21.2	-	37.0
	male	-	10.1	-	12.4	-	23.6	-	35.1
	female	-	14.6	-	15.6	-	19.5	-	38.7
Total Misconcept	tot	-	3.6	-	0.0	-	8.8	-	6.7
	male	-	5.1	-	0.0	-	7.9	-	8.8
	female	-	2.2	-	0.0	-	9.4	-	4.8
Partial Knoweldge	tot	-	92.9	-	96.6	-	95.9	-	98.3
	male	-	89.9	-	94.4	-	95.5	-	96.5
	female	-	95.5	-	98.9	-	96.1	-	100
Partial Misconcept	tot	-	56.5	-	66.5	-	78.8	-	82.4
	male	-	59.5	-	62.9	-	77.5	-	82.5
	female	-	53.9	-	70.0	-	79.7	-	82.3
Misconcept	tot	-	100	-	100	-	100	-	100
	male	-	100	-	100	-	100	-	100
	female	-	100	-	100	-	100	-	100
