# Supplementary material for: Elimination testing with adapted scoring reduces guessing and anxiety in multiple-choice assessments, but does not increase grade average in comparison with negative marking
Source: PLoS One. 2018 Oct 2;13(10):e0203931. doi: 10.1371/journal.pone.0203931 (PMC6168139; doi:10.1371/journal.pone.0203931)
Supplement: S5 Table — The knowledge levels are defined in Table 3. Partial knowledge (PK) is an aggregation of partial knowledge 1 (PK1), partial knowledge 2 (PK2), and partial knowledge 3 (PK3). PMI is an aggregation of partial misconcept 2 (PM2), partial misconcept 3 (PM3), and total misconcept (TM). Misconcept (M) is an aggregation of partial misconcept 1 (PM1), partial misconcept 2 (PM2), partial misconcept 3 (PM3), and total misconcept (TM). M = male, F = female. (PDF) [file pone.0203931.s009.pdf]

S6 Table. Average number of questions in a particular knowledge level per student in a multiple choice exam. The knowledge levels are defined in Table 3. Partial knowledge (PK) is an aggregation of partial knowledge 1 (PK1), partial knowledge 2 (PK2), and partial knowledge 3 (PK3). PMI is an aggregation of partial misconcept 2 (PM2), partial misconcept 3 (PM3), and total misconcept (TM). Misconcept (M) is an aggregation of partial misconcept 1 (PM1), partial misconcept 2 (PM2), partial misconcept 3 (PM3), and total misconcept (TM). M = male, F = female.

		Pediatrics				Gynaecology			
		T1		T2		T1		T2	
		NM	ETA	NM	ETA	NM	ETA	NM	ETA
FK	tot	30.55(3.59)	28.73(4.25)	26.91(4.40)	26.64(4.33)	58.68(9.01)	53.02(11.08)	57.09(9.12)	48.72(12.04)
	M	30.02(3.41)	28.44(4.66)	26.16(4.72)	26.56(4.74)	58.14(9.61)	51.64(11.71)	56.19(10.48)	46.04(12.73)
	F	30.91(3.67)	28.99(3.83)	27.60(3.97)	26.72(3.87)	59.16(8.41)	53.98(10.51)	57.98(7.43)	51.19(10.80)
PK1	tot	-	3.21(2.57)	-	3.30(2.22)	-	6.98(4.64)	-	7.80(4.42)
	M	-	3.48(2.88)	-	3.34(2.45)	-	7.30(4.93)	-	8.12(5.08)
	F	-	2.97(2.23)	-	3.27(1.97)	-	6.76(4.42)	-	7.50(3.69)
PK2	tot	-	1.03(1.18)	-	1.17(1.36)	-	2.53(2.64)	-	2.51(2.35)
	M	-	0.91(1.24)	-	0.96(1.15)	-	2.56(2.45)	-	2.58(2.57)
	F	-	1.13(1.10)	-	1.38(1.52)	-	2.52(2.76)	-	2.45(2.12)
PK3	tot	-	0.41(0.78)	-	0.37(0.68)	-	0.98(1.49)	-	1.40(2.07)
	M	-	0.28(0.64)	-	0.31(0.61)	-	1.04(1.55)	-	1.33(1.97)
	F	-	0.53(0.88)	-	0.43(0.73)	-	0.94(1.45)	-	1.47(2.15)
NK	tot	2.25(2.36)	0.57(1.09)	3.05(2.74)	0.80(1.08)	7.55(5.88)	2.65(3.52)	7.95(6.44)	3.75(4.91)
	M	2.40(2.41)	0.42(0.89)	3.28(2.73)	0.78(0.96)	6.59(5.68)	2.81(3.67)	7.37(6.12)	4.25(4.90)
	F	2.14(2.32)	0.71(1.23)	2.84(2.74)	0.83(1.19)	8.40(5.92)	2.55(3.41)	8.52(6.70)	3.29(4.87)
PM1	tot	7.20(2.93)	4.96(2.37)	10.04(4.04)	6.37(2.79)	13.77(6.04)	11.49(4.92)	14.96(6.35)	12.73(7.12)
	M	7.57(2.88)	5.18(2.68)	10.56(3.83)	6.56(3.12)	15.27(6.33)	12.09(4.88)	16.44(7.40)	14.12(8.11)
	F	6.95(2.93)	4.76(2.03)	9.56(4.16)	6.18(2.41)	12.44(5.44)	11.07(4.91)	13.50(4.66)	11.45(5.78)
PM2	tot	-	0.90(1.19)	-	1.17(1.29)	-	1.89(2.24)	-	2.50(2.29)
	M	-	1.11(1.41)	-	1.34(1.48)	-	2.04(2.70)	-	2.95(2.35)
	F	-	0.72(0.90)	-	1.01(1.05)	-	1.78(1.84)	-	2.08(2.15)
PM3	tot	-	0.15(0.42)	-	0.17(0.44)	-	0.35(1.03)	-	0.52(0.86)
	M	-	0.13(0.40)	-	0.16(0.45)	-	0.43(1.15)	-	0.53(0.90)
	F	-	0.17(0.43)	-	0.18(0.44)	-	0.30(0.93)	-	0.52(0.82)
TM	tot	-	0.04(0.19)	-	0.00(0.00)	-	0.10(0.33)	-	0.07(0.25)
	M	-	0.05(0.22)	-	0.00(0.00)	-	0.08(0.27)	-	0.09(0.28)
	F	-	0.02(0.15)	-	0.00(0.00)	-	0.11(0.36)	-	0.05(0.21)
PK	tot	-	4.65(3.38)	-	4.84(3.21)	-	10.50(7.36)	-	11.71(6.46)
	M	-	4.67(3.61)	-	4.61(3.37)	-	10.91(7.41)	-	12.04(7.05)
	F	-	4.63(3.15)	-	5.08(3.03)	-	10.21(7.31)	-	11.42(5.85)
PMI	tot	-	1.09(1.35)	-	1.34(1.38)	-	2.34(3.03)	-	3.08(2.74)
	M	-	1.29(1.61)	-	1.49(1.60)	-	2.55(3.65)	-	3.56(2.83)
	F	-	0.91(1.05)	-	1.19(1.10)	-	2.20(2.49)	-	2.65(2.57)
M	tot	-	6.05(2.62)	-	7.71(2.96)	-	13.83(5.72)	-	15.82(8.29)
	M	-	6.47(2.90)	-	8.06(3.30)	-	14.64(6.00)	-	17.68(8.88)
	F	-	5.67(2.28)	-	7.37(2.53)	-	13.27(5.45)	-	14.10(7.29)
