# Supplementary material for: Elimination testing with adapted scoring reduces guessing and anxiety in multiple-choice assessments, but does not increase grade average in comparison with negative marking
Source: PLoS One. 2018 Oct 2;13(10):e0203931. doi: 10.1371/journal.pone.0203931 (PMC6168139; doi:10.1371/journal.pone.0203931)
Supplement: S1 File — (PDF) [file pone.0203931.s010.pdf]

**S10 File. Preference questionnaire response analyses.**

This appendix reports on the influence of student characteristics on their preference for negative marking or elimination testing with adapted scoring. To investigate this influence this analysis focuses on the replies of students on question 42 of the questionnaire "After taking all aspects into consideration, I prefer elimination testing with adapted scoring" (preferenceETA).

To this end the following regression model was built:

$$preferenceETA \sim scoreETA + courseETA + gender + EM + GPA * courseETA + scoreETA * \ gender + scoreETA * EM \ + scoreETA * courseETA * EM,$$

where courseETA indicates for which course the students had elimination testing with adapted scoring and hereby, due to the crossed test design, in which master they were. EM abbreviates the examination moment and GPA abbreviates the grade point average.

The regression model is weak (adjusted $R^2 = 0.0001$, degrees of freedom 175, residual error 1.234), so the preference of students is only weakly related to student characteristics and exam score itself. The regression analysis (Table 1) shows that the preference for elimination testing with adapted scoring does not significantly depend on any of the independent variables, hereby showing there is no significant influence of gender and exam score on the preference for elimination testing with adapted scoring in comparison to negative marking.

**Table 1 Results of multiple linear regression for predicting scoring method preference (prefer elimination testing with adapted scoring).**

| | ***β*** | ***t*()** | ***p*** |
|---|---|---|---|
| Intercept | 3.019 (0.787) | 3.838 | $0.000^{***}$ |
| scoreETA | 0.018(0.059) | 0.312 | $0.755^{ns}$ |
| courseETA [pediatrics] | 1.156 (1.109) | 1.042 | $0.299^{ns}$ |
| gender [F] | -1.449 (0.971) | -1.493 | $0.137^{ns}$ |
| EM [T2] | 0.401 (1.474) | 0.272 | $0.786^{ns}$ |
| scoreETA *courseETA | -0.086 (0.078) | -1.098 | $0.274^{ns}$ |
| scoreETA *gender | -0.090 (0.069) | 1.306 | $0.193^{ns}$ |
| scoreETA * EM | 0.012 (0.117) | 0.101 | $0.919^{ns}$ |
| scoreETA *courseETA*EM | - | - | - |

The table shows the regression coefficients (β) and the standard deviation between brackets the question "After taking all aspects into consideration, I prefer elimination testing with adapted scoring" (N=683, $R^2$=0.0001). courseETA = course that was assessed using elimination testing with adapted scoring, EM = examination moment. Superscripts indicate levels of significance using the following coding: ns $p > 0.05$; * $p < 0.05$; ** $p < 0.01$; *** $p < 0.001$.

If the GPA, operationalizing ability, is used for the regression rather than the exam score (scoreETA) the following model is obtained:

$$preferenceETA \sim GPA + courseETA + gender + EM + GPA * courseETA + GPA * gender + GPA * EM + GPA * courseETA * EM.$$

Again, the regression model is weak (adjusted $R^2 = 0.06$, degrees of freedom 175, residual error 1.193). The regression analysis (Table 2) shows that the student preference for elimination testing with adapted scoring however depends on the course (and thus the master), and that there is an interaction effect between the course and GPA. In detail: firstly, if elimination testing with adapted scoring was used for pediatrics (2nd master students), students prefer elimination testing with adapted scoring more than if elimination testing with adapted scoring was used for GO (1st master students); secondly if elimination testing with adapted scoring was used for pediatrics (1st master students) students with higher GPA have a lower preference for elimination testing with adapted scoring but if elimination testing with adapted scoring was used for Gynaecology (2nd master students), there is no influence of GPA on the preference (Fig. ***1***). One possible explanation of the lower preference of high ability students for elimination testing with adapted scoring in pediatrics is that high ability students doubt less and therefore less often use the possibility of elimination testing with adapted scoring to express partial knowledge, while they still having to color each alternative (disadvantage of elimination testing with adapted scoring over negative marking).

**Table 2 Results of multiple linear regression for predicting scoring method preference (prefer elimination testing with adapted scoring).**

The table shows the regression coefficients (β) and the standard deviation between brackets the question "After taking all aspects into consideration, I prefer elimination testing with adapted scoring" (N=683, $R^2$=0.0646). courseETA = course that was assessed using elimination testing with adapted scoring, EM = examination moment. Superscripts indicate levels of significance using the following coding: ns $p > 0.05$; * $p < 0.05$; ** $p < 0.01$; *** $p < 0.001$.

| | ***β*** | ***t*()** | ***p*** |
|---|---|---|---|
| Intercept | 3.743 (1.248) | 2.998 | 0.003** |
| GPA | -0.007(0.018) | -0.402 | 0.688 ns |
| courseETA [pediatrics] | -4.125 (1.556) | 2.650 | 0.009** |
| gender [female] | -1.733 (1.464) | -1.184 | 0.238ns |
| EM [T2] | -0.277 (2.548) | -0.109 | 0.913ns |
| GPA*courseETA | -0.057(0.021) | -2.643 | 0.009** |
| GPA*gender | -0.021 (0.020) | 1.032 | 0.304ns |
| GPA*EM | 0.012 (0.037) | 0.313 | 0.755ns |
| GPA*courseETA*EM | - | - | - |

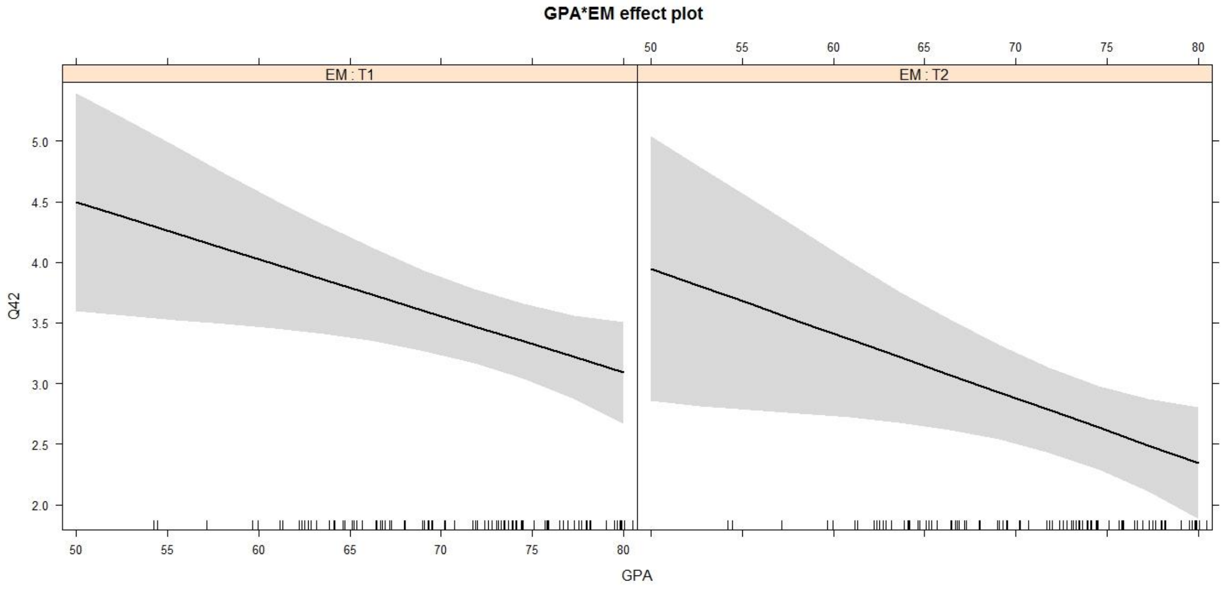


***Fig. 1: Effect plot of GPA and examination moment for pediatrics on the preference of students for elimination testing with adapted scoring.***
